# Genetic mapping of microbial and host traits reveals production of immunomodulatory lipids by *Akkermansia muciniphila* in the murine gut

**DOI:** 10.1038/s41564-023-01326-w

**Published:** 2023-02-09

**Authors:** Qijun Zhang, Vanessa Linke, Katherine A. Overmyer, Lindsay L. Traeger, Kazuyuki Kasahara, Ian J. Miller, Daniel E. Manson, Thomas J. Polaske, Robert L. Kerby, Julia H. Kemis, Edna A. Trujillo, Thiru R. Reddy, Jason D. Russell, Kathryn L. Schueler, Donald S. Stapleton, Mary E. Rabaglia, Marcus Seldin, Daniel M. Gatti, Gregory R. Keele, Duy T. Pham, Joseph P. Gerdt, Eugenio I. Vivas, Aldons J. Lusis, Mark P. Keller, Gary A. Churchill, Helen E. Blackwell, Karl W. Broman, Alan D. Attie, Joshua J. Coon, Federico E. Rey

**Affiliations:** 1 Department of Bacteriology, University of Wisconsin-Madison, Madison, WI USA; 2 Department of Chemistry, University of Wisconsin-Madison, Madison, WI USA; 3 IMol Polish Academy of Sciences, Warsaw, Poland; 4 ReMedy International Research Agenda Unit, IMol Polish Academy of Sciences, Warsaw, Poland; 5 Department of Biomolecular Chemistry, University of Wisconsin-Madison, Madison, WI USA; 6 Morgridge Institute for Research, Madison, WI USA; 7 Department of Biochemistry, University of Wisconsin-Madison, Madison, WI USA; 8 Departments of Microbiology, Immunology and Molecular Genetics, and Human Genetics, University of California, Los Angeles, Los Angeles, CA USA; 9 Department of Medicine, University of California, Los Angeles, Los Angeles, CA USA; 10 The Jackson Laboratory, Bar Harbor, ME USA; 11 Department of Chemistry, Indiana University, Bloomington, IN USA; 12 Department of Biostatistics and Medical Informatics, University of Wisconsin-Madison, Madison, WI USA

**Keywords:** Genetic association study, Genetic linkage study, Metagenomics, Lipidomics, Lipids

## Abstract

The molecular bases of how host genetic variation impacts the gut microbiome remain largely unknown. Here we used a genetically diverse mouse population and applied systems genetics strategies to identify interactions between host and microbe phenotypes including microbial functions, using faecal metagenomics, small intestinal transcripts and caecal lipids that influence microbe–host dynamics. Quantitative trait locus (QTL) mapping identified murine genomic regions associated with variations in bacterial taxa; bacterial functions including motility, sporulation and lipopolysaccharide production and levels of bacterial- and host-derived lipids. We found overlapping QTL for the abundance of *Akkermansia muciniphila* and caecal levels of ornithine lipids. Follow-up in vitro and in vivo studies revealed that *A. muciniphila* is a major source of these lipids in the gut, provided evidence that ornithine lipids have immunomodulatory effects and identified intestinal transcripts co-regulated with these traits including *Atf3*, which encodes for a transcription factor that plays vital roles in modulating metabolism and immunity. Collectively, these results suggest that ornithine lipids are potentially important for *A. muciniphila–*host interactions and support the role of host genetics as a determinant of responses to gut microbes.

## Abstract (web summary)

Systems genetics reveals interactions between host and microbial phenotypes in the murine gut, including a role for *Akkermansia muciniphila* in the production of immunomodulatory ornithine lipids.

## Main

The gut microbiome plays fundamental roles in mammalian physiology and human health^1–3^. Environmental exposures and host genetic variation modulate gut microbiota composition^4–6^ and contribute to the large degree of interpersonal variation observed in human gut microbial communities. Recent advances in sequencing technologies and analytical pipelines have fuelled progress in our understanding of the impact of host genetics and the gut microbiome on health. Population studies have revealed host genetic-gut microbial trait associations in human^7–11^ and mouse cohorts^12,13^. Additionally, studies leveraging host genetic information and Mendelian randomization have highlighted connections between the gut microbiome and other molecular complex traits including faecal levels of short-chain fatty acids^14^, plasma proteins^15^ and ABO histo-blood group type^16^ in humans. However, most of these studies have focused on microbial organismal composition and there is currently a major gap in our understanding of the impact of host genetic variation on the functional capacity of the gut microbiome.

Microbial metabolites are critical nodes of communication between microbes and the host. These include small molecules derived from dietary components (for example, Trimethylamine N-oxide)^17^ or de novo synthesized by microbes such as vitamins^18^ and lipids^19^. Lipids including eicosanoids, phospholipids, sphingolipids and fatty acids act as signalling molecules to control many cellular processes^20–22^. Gut microbes not only modulate absorption of dietary lipids via regulation of bile acid production and metabolism but are also a major source of lipids and precursor metabolites for lipids produced by the host^23,24^. Bacterial cell membrane-associated lipids are also important for microbe–host interactions^19,25^, although our understanding of their roles in these dynamics is only emerging for gut bacteria.

Defining the general principles that govern microbe–host interactions in the gut ecosystem is a daunting task. Systems genetic studies can generate hypotheses that invoke processes and molecules that have no precedent, which can be used for the identification of genes, pathways and networks underlying these interactions. To investigate the connections between gut microbes, intestinal lipids and host genetic variation, we leveraged the Diversity Outbred (DO) mouse cohort, a genetically diverse population derived from eight founder strains: C57BL/6J (B6), A/J (A/J), 129S1/SvImJ (129), NOD/ShiLtJ (NOD), NZO/HLtJ (NZO), CAST/EiJ (CAST), PWK/PhJ (PWK) and WSB/EiJ (WSB)^26,27^. These eight strains harbour distinct gut microbial communities and exhibit disparate metabolic responses to diet-induced metabolic disease^28^. The DO population is maintained by an outbreeding strategy aimed at maximizing the power and resolution of genetic mapping. We characterized the faecal metagenome, intestinal transcriptome and caecal lipidome in DO mice and performed quantitative trait locus (QTL) analysis to identify host genetic loci associated with these traits. We integrated microbiome QTL (mbQTL) and caecal lipidome QTL (clQTL) to uncover microbe–lipid associations and identified candidate genes expressed in the distal small intestine associated with these co-mapping traits. These datasets represent a valuable resource for interrogating the molecular mechanisms underpinning interactions between the host and the gut microbiome.

## Results

### Gut microbial features are associated with host genetics

**Extended Data Fig. 1:**
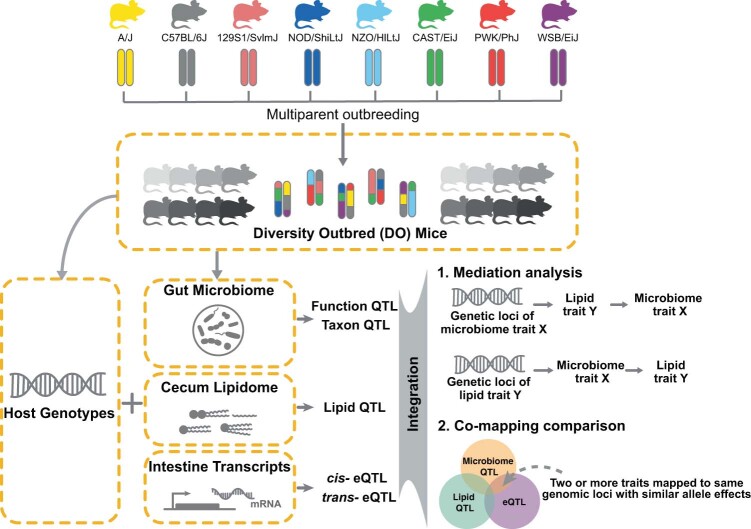
Overview of the study. Fecal metagenomes (n = 264 animals), caecal lipidomes (n = 381 animals) and distal small intestine transcriptomes (n = 234 animals) were generated from Diversity Outbred mice. Quantitative trait loci (QTL) analysis identified genomic regions associated with variations in bacterial taxa, bacterial functions, levels of bacterial- and host-derived lipids and small intestine transcript levels. Mediation analysis and co-mapping comparisons were used to identify causal links between traits.

**Extended Data Fig. 2:**
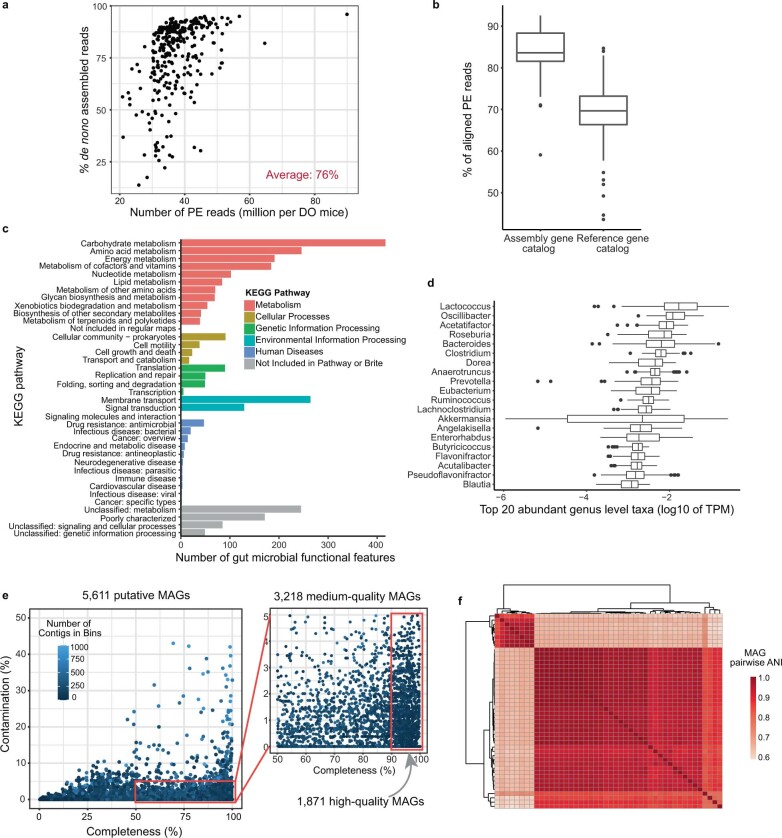
DO metagenomic analysis. **a**, Average percent of assembled reads across all samples. **b**, Comparison of percent of reads mapping to our generated assembly *vs*. public database (n = 297 animals). **c**, Microbial functions detected for KEGG pathways across all metagenomes. KEGG Orthology (KO) numbers were identified by annotating predicted ORFs to the KEGG database. **d**, Top 20 gut microbial genera detected across all DO mice (n = 264 animals). **e**, Quality of metagenome-assembled genomes. **f**, Two variants of *A. muciniphila* MAGs detected in the DO mice. Box and whisker plots denote the interquartile range, median and spread of points within 1.5 times the interquartile range, data beyond the end of the whiskers are plotted individually. Source data

We characterized the faecal microbiome from 264 DO mice fed a high-fat high-sucrose (HF/HS) diet for ~22 weeks (Extended Data Fig. 1). We and others previously showed that this diet elicits a wide range of metabolic responses in the eight founder strains that are associated with microbiome changes, and identified loci associated with variation in abundance of bacterial taxa in the gut^28,29^; here we examine the role of host genetics in influencing gut microbiome traits with a focus on gut bacterial functions. Metagenomic analysis revealed ~1.9 million unique predicted microbial open reading frames (that is, metagenes), 2,803 bacterial functions (KEGG orthologues, KOs) and 187 bacterial taxa across all mice. We also performed metagenomic binning to obtain metagenome-assembled genomes (MAGs), corresponding to species-level bacterial genomes (Extended Data Fig. 2, Supplementary Tables 1–4 and Supplementary Note 1).

**Fig. 1:**
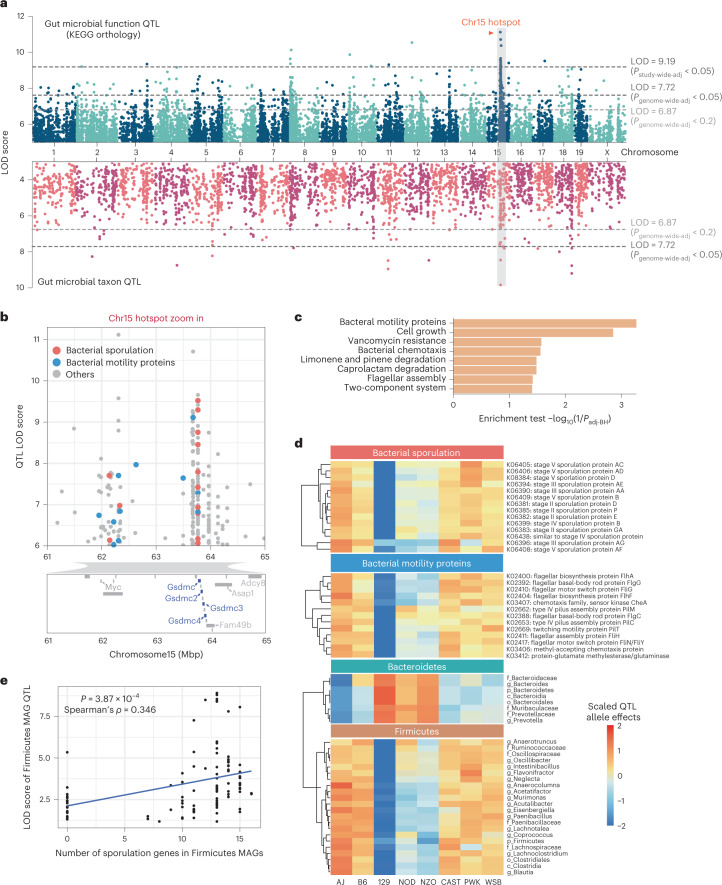
Genetic architecture of QTL for microbial traits in the DO mouse cohort. **a**, QTL mapping results for 2,803 gut microbial KO function traits (top panel) and 187 bacterial taxa traits (bottom panel) using sex, days on diet and cohort as covariates. Each dot represents a QTL on the mouse genome for a given trait. Dashed lines represent significance thresholds for QTL determined by permutation tests (LOD > 9.19, *P*_study-wide-adj_ < 0.05; LOD > 7.72, *P*_genome-wide-adj_ < 0.05; LOD > 6.87, *P*_genome-wide-adj_ < 0.2). QTL hotspot at Chromosome 15 is highlighted by grey shading and orange colour text. **b**, Gut microbiome QTL hotspot on Chr15 has multiple bacterial sporulation and motility functions mapping to it. Protein coding genes are displayed for Chr15: 61–65 Mbp region, Gasdermin genes are highlighted in blue. **c**, Enrichment analysis (Fisher’s exact test) for functions mapping at hotspot on Chr15. **d**, QTL for microbial functions that mapped to Chromosome 15 hotspot had negative 129S1/SvImJ allele effects. QTL for Firmicutes mapping to Chromosome 15 hotspot had negative 129S1/SvImJ allele effects, whereas QTL for Bacteroidetes mapping to this locus had positive 129S1/SvImJ allele effects. **e**, Spearman correlation analysis between the number of sporulation KOs detected in Firmicutes MAGs mapping at Chromosome 15 QTL hotspot and the LOD scores for these MAGs (*P* = 3.87 × 10^−3^, Spearman’s *ρ* = 0.346). Source data

**Extended Data Fig. 3:**
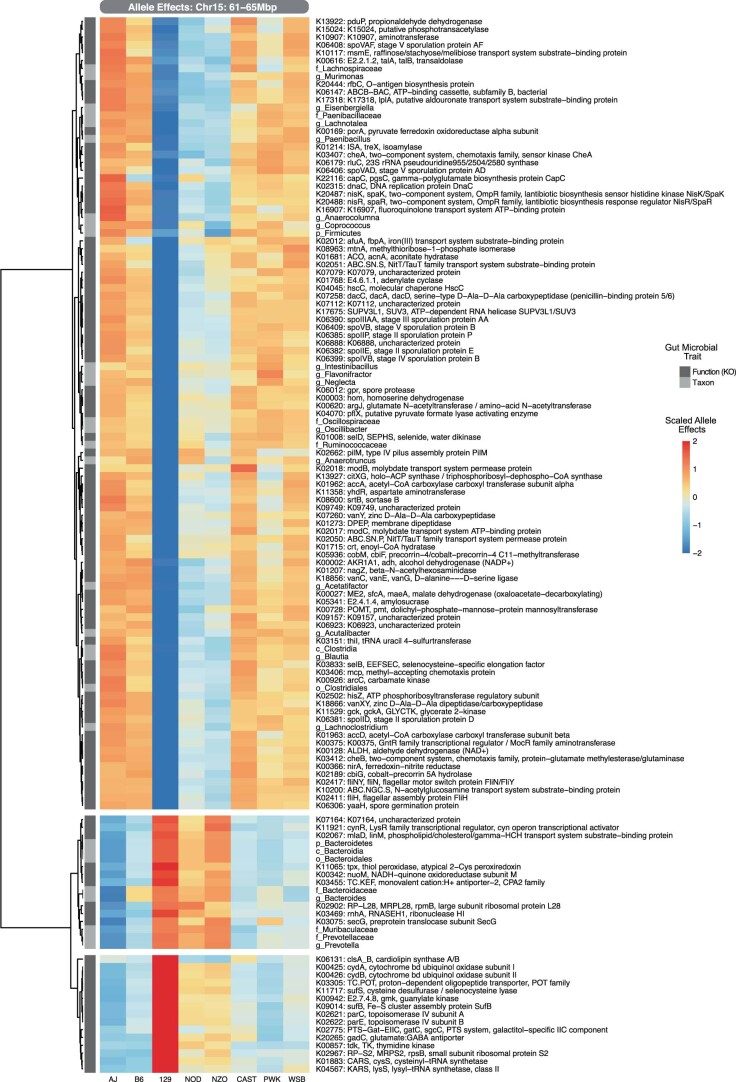
DO gut microbiome QTL hotspot at Chr15: 61–65Mbp. Founder allele effects of KO and taxa trait QTL at Chr15 hotspot (LOD > 6). Source data

We next used QTL analysis to identify regions of the mouse genome associated with the abundance of these traits. We detected 760 associations for KOs (logarithm of odds (LOD) > 6.87, *P*_genome-wide-adj_ < 0.2), 200 of which were genome-wide significant (LOD > 7.72, *P*_genome-wide-adj_ < 0.05) and 45 associations for bacterial taxa (LOD > 6.87*, P*_genome-wide-adj_ < 0.2), 15 of which were genome-wide significant (LOD > 7.72, *P*_genome-wide-adj_ < 0.05) (Fig. 1a and Supplementary Tables 5 and 6). We identified a QTL hotspot on chromosome 15 at 63–64 Mbp; this genomic region was associated with 154 microbial traits with LOD score > 6 (Supplementary Table 7). We estimated DO founder allele effects as best linear unbiased predictors for the traits that mapped to this locus. Among these, we detected two clear groups of traits that exhibited opposite allele effects: a group of KOs and taxa showing positive association with the 129 allele, and another group of KOs and taxa that were negatively associated with the 129 allele (Extended Data Fig. 3). As detailed below, the two most abundant gut bacterial phyla, Firmicutes and Bacteroidetes, mapped to this locus with opposite allele effects.

**Extended Data Fig. 4:**
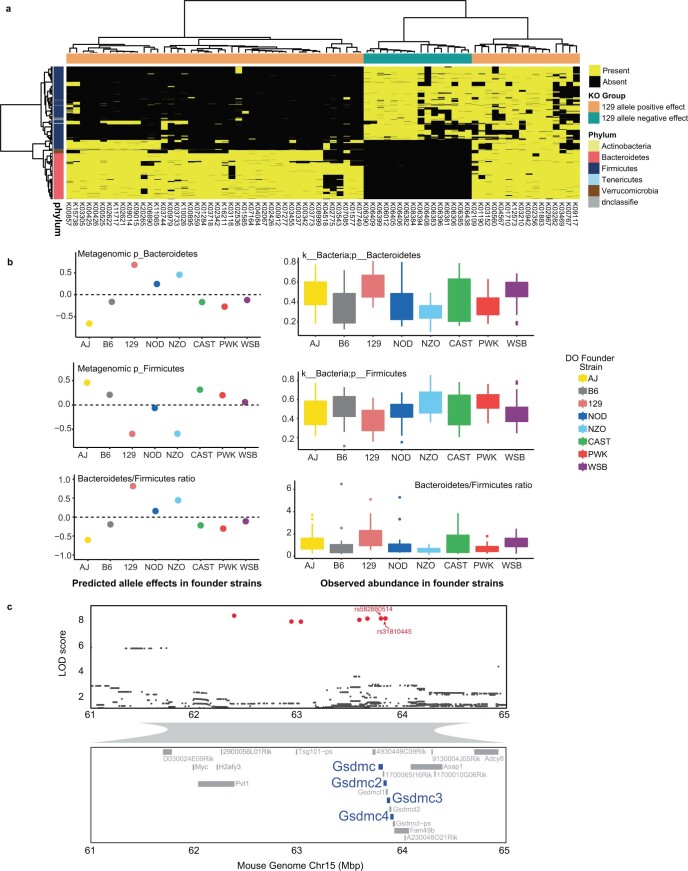
DO gut microbiome QTL hotspot and SNP associations. **a**, Presence/absence of KOs that mapped to Chr15 hotspot across all MAGs. Sporulation functions were not detected in Bacteroidetes. **b**, Estimated founder allele effects for Bacteroidetes and Firmicutes, and Bacteroidetes/Firmicutes ratio (left panel). Observed abundance of Bacteroidetes Firmicutes and Bacteroidetes/Firmicutes ratio in founder strains as determined by Kemis et al. (right panel, n = 9-12 animals/founder strain). **c**, SNPs significantly associated with these traits in Chr15 hotspot include two intron SNPs in *Gsdmc* and *Gsdmc2* genes. Box and whisker plots denote the interquartile range, median and spread of points within 1.5 times the interquartile range, data beyond the end of the whiskers are plotted individually. Source data

Pathway enrichment analysis showed that bacterial ‘motility proteins’ and ‘cell growth’ functional categories were significantly enriched in the group of KOs associated most strongly with 129 alleles (Fig. 1b,c). More specifically, abundances of 14 sporulation functions were negatively associated with 129 alleles (Fig. 1d). Further investigation of the KO distribution across all MAGs revealed that all bacterial sporulation KOs were only present in MAGs belonging to Firmicutes, whereas most of KOs that showed positive 129 allele effects were present in MAGs belonging to Bacteroidetes (Extended Data Fig. 4a). To assess whether the allele effects observed from QTL mapping corresponded to the trait patterns in the DO founder strains, we examined previously published 16S ribosomal RNA gene data from age-matched mice from the eight founder strains, also fed an HF/HS diet^13^. Consistent with these findings, we found that the 129 mouse strain had higher levels of Bacteroidetes and the highest Bacteroidetes/Firmicutes ratio (Extended Data Fig. 4b). Interestingly, we detected a significant positive correlation between the number of sporulation KOs in Firmicutes MAGs mapping at this locus and the LOD scores for these MAGs (Fig. 1e). Importantly, Firmicutes MAGs commonly detected in our dataset that do not contain sporulation KOs (for example, *Lactobacillus*, *Lactococcus*) did not exhibit significant association to this QTL. These results support the notion that host genetic variation affects gut community structure in part by modulating the abundance of sporulating bacteria.

Single nucleotide polymorphism (SNP) association analysis within the Chr15 QTL hotspot identified six significant SNPs: two intron variants, SNP rs582880514 in the *Gsdmc* gene and SNP rs31810445 in the *Gsdmc2* gene, both with LOD scores of 8.0; four SNPs that were intergenic variants (Extended Data Fig. 4c). Gasdermins (Gsdm) are a family of pore-forming proteins that cause membrane permeabilization and pyroptosis^30^, an inflammatory form of programmed cell death that is triggered by intra- and extracellular pathogens^31^. These results indicate that host genetic variation in Gsdmc/Gsdmc2 is associated with abundance of gut bacterial functions and raises the hypothesis that these host proteins could modulate the abundance of bacterial taxa harbouring motility and/or sporulation functions.

### Caecal lipids are associated with gut microbes and host genetics

**Fig. 2:**
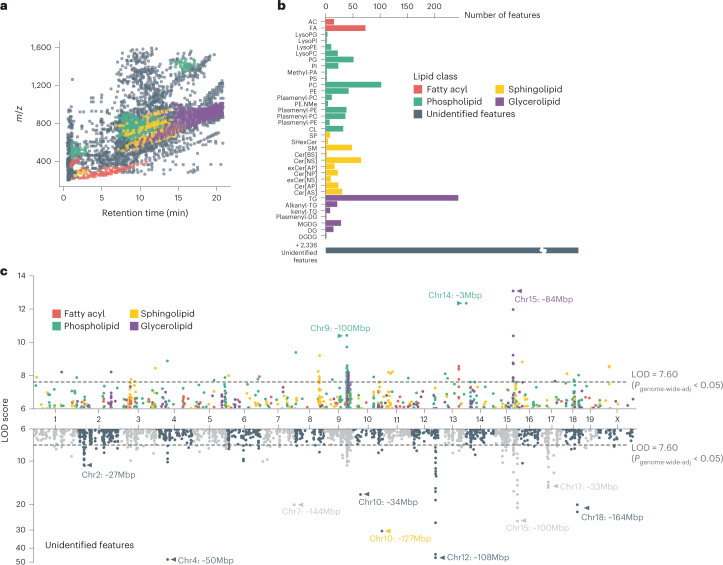
Genetic architecture of the caecal lipidome in DO mice. **a**, A total of 3,384 caecal lipid features were quantified across 381 DO mice, 1,048 of which were identified as lipids from four major classes. Each dot represents a caecal lipid feature. Features of each class occupied characteristic regions in the *m*/*z* – RT space. **b**, Identified lipids belonged to 35 lipid subclasses, with bacteria-associated PG and MGDG as common subclasses. **c**, A total of 3,964 suggestive caecal lipid QTL (LOD > 6, *P*_genome-wide-adj_ < 0.2) and 12 QTL hotspots were identified. Hotspots are marked with arrows and the corresponding genomic locus indicated. Dashed lines represent significance thresholds for QTL as determined by permutation tests (LOD > 7.60, *P*_genome-wide*-*adj_ < 0.05). Of the identified lipids, 68.2% showed a total of 1,162 QTL (top panel), while a similar portion of 70.1% of unidentified features contributed 2,802 QTL (bottom panel). RT, retention time. For lipid class abbreviations, see Supplementary Table 16. Source data

**Extended Data Fig. 5:**
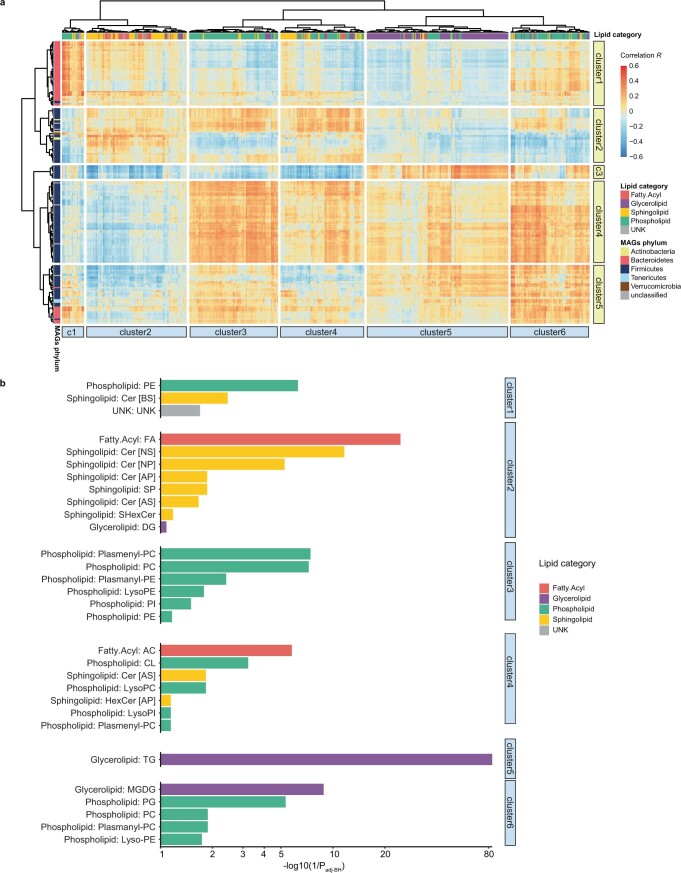
Correlation between gut bacterial MAGs and caecal lipids. **a**, Heatmap showing two-sided Spearman correlation coefficients between the abundance of MAGs and caecal lipid levels across DO mice. Bacterial MAGs were clustered into five groups whereas caecal lipids were clustered into six groups. **b**, Enrichment of the lipid classes for each caecal lipid clusters. Fisher’s exact test was used and Benjamini-Hochberg for multiple tests correction. Source data

We employed a broad discovery strategy to agnostically detect lipid actors potentially relevant to gut microbiome–host interactions. We used liquid chromatography coupled with tandem mass spectrometry (LC–MS/MS) to characterize the caecal lipidome of 381 DO mice, including all mice used for the metagenomic analysis. We identified 1,048 lipid species representing 35 lipid classes (Fig. 2a,b) and the four major lipid categories: (1) fatty acyls, (2) phospholipids, (3) sphingolipids and (4) glycerolipids. The highest numbers of lipids were recorded for the classes of triglycerides (TG) and phosphatidylcholines (PC), species known to be abundant in the mammalian host^32^. Of the 3,384 lipid species detected in DO caecum, 547 (16.2%) were detected at higher levels in the caecum of conventionally raised mice compared with caecum of germ-free animals (fold-change >10-fold, adjusted *P* < 0.05). Phosphatidylglycerols (PG), for example, which represent the second largest phospholipid class in our data, are known to be a major component of the bacterial lipidome^33^. In mammals, on the other hand, PG are only a minor component. Similarly, among glycerolipids, monogalactosyldiacylglycerols (MGDG) account for the second highest number of lipids detected in this class. While they are found at high levels in bacteria and plants, these lipids are only minor components of animal tissue^34^. These findings suggest that our analysis of the caecal lipidome captures components of the host and the gut microbiome. Correlation analysis between MAGs and caecal lipids abundance, plus comparison of the caecal lipidome of conventionally raised vs germ-free mice identified taxa that potentially modulate the abundance of lipids in the gut (Extended Data Fig. 5a,b, Supplementary Tables 8–10 and Supplementary Note 2). Furthermore, QTL mapping identified 399 significant QTL associations for caecal lipid features (LOD > 7.60, *P*_genome-wide-adj_ < 0.05) (Fig. 2c, Supplementary Table 11 and Supplementary Note 3). Altogether these associations provide a wealth of information offering potential molecular descriptors of the genetic regulation of the microbiome.

### Mediation analysis reveals bacteria–caecal lipids connections

**Fig. 3:**
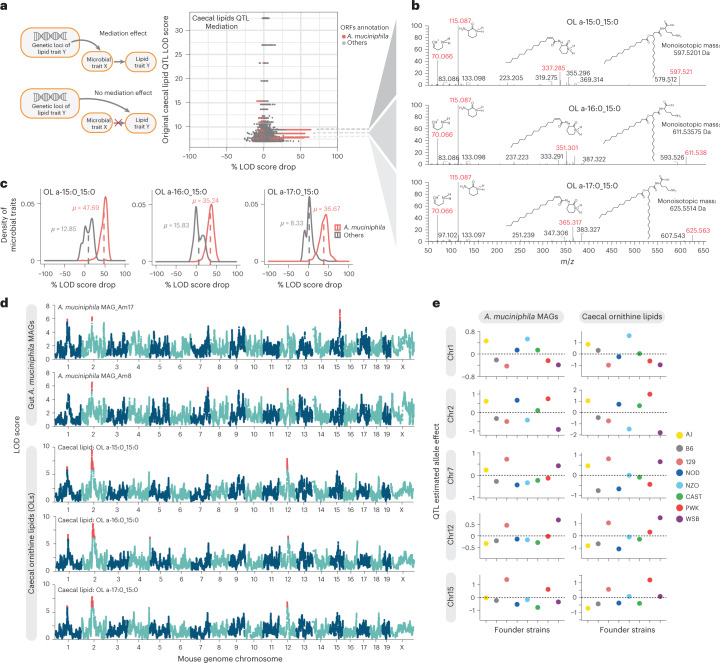
Mediation analysis revealed potential causal relationship between *A. muciniphila* and OL. **a**, Illustration of Mediation effect model and Null model. Each dot in the scatterplot represents the result of the mediation test for a gut microbial metagene–caecal lipid feature pair; *x* axis shows the drop in QTL LOD score for caecal lipid features when adding gut microbial metagenes as covariates to the caecal lipid QTL model; *y* axis shows the original QTL LOD score for each caecal lipid. Dots with the same *y* axis value represent the mediation test of individual metagenes with one caecal lipid feature. A high QTL LOD score drop represents a significant mediation effect of the gut microbial feature to the caecal lipid feature. Association of three unknown caecal lipid features with the host genome was impacted by *A. muciniphila* genes. This is depicted as multiple red dots (many dots appear as lines) showing significant mediation effects. **b**, Three lipid features mediated by *A. muciniphila* genes were identified as ornithine lipids. The dashed lines connecting **a** and **b** point to the fragmentation patterns of identified ornithine lipids, as shown by the *m*/*z* values; key fragments are shown in red colour together with their respective chemical structures. **c**, Distribution of LOD score drop when adding individual *A. muciniphila* genes as covariates (Mediation model) or adding individual genes not from *A. muciniphila* as covariates (Null model) for three identified ornithine lipids. **d**, Three ornithine lipids species QTL co-mapped at five loci (Chromosome 1, Chromosome 2, Chromosome 7, Chromosome 12, Chromosome 15) with *A. muciniphila* MAGs QTL.QTL with LOD > 5.5 are highlighted by red colour. **e**, Founder allele effects for *A. muciniphila* MAGs and caecal OL were estimated in the DO population from the founder strain coefficients observed for the corresponding QTL at each locus from **d**. Source data

To identify causal links between gut microbial traits and caecal lipid traits, we performed mediation analysis between individual gut microbial metagenes and lipid features that co-map (Methods). Mediation analysis seeks to determine whether a QTL has separate effects on two traits, or if it affects one trait through its effect on another trait, in which case the intermediate trait is called a mediator. Figure 3a shows gut microbial metagenes mediating the QTL effect on a caecal lipid trait. We reasoned that if a microbial trait influenced a caecal lipid that was independent from the caecal lipid’s QTL, its inclusion as a covariate would be unlikely to affect the caecal lipid QTL signal significantly. However, for microbial traits that mediate the QTL effect on the caecal lipid, there would be a large drop in the original caecal lipid QTL LOD score. Interestingly, we found three caecal lipid features with QTL that were mediated by microbial metagenes. Most of these mediating microbial traits were genes belonging to the bacterium *Akkermansia muciniphila*. It is important to note that the direction of the causal effect between microbial trait and caecal lipid cannot be directly inferred from the data. These results suggest that *A. muciniphila* levels and the abundance of these lipid species in the gut are modulated by the same loci and that the two traits are potentially connected (Fig. 3b,c).

We further tested whether these caecal lipids and *A. muciniphila* mapped to the same loci. Mapping of the 46 reconstructed *A. muciniphila* MAGs to the host genome revealed multiple QTL including Chr1: 92.9 Mbp, Chr2: 79.4 Mbp, Chr7: 129.8 Mbp, Chr12: 59.4 Mbp, and Chr15: 75.9 Mbp (Fig. 3d). Interestingly, the three caecal lipids also showed QTL at the same loci and exhibited similar founder allele effect patterns (Fig. 3e). These founder allele effects on *A. muciniphila* abundance are consistent with a previous study of gut bacterial abundance in the DO founder strains^13^. Although these lipid features were not initially identified by our lipidomic analysis pipeline, they appeared to be closely related to each other. Further analysis of their fragmentation spectra suggested that these unidentified features were ornithine lipids (OL) (Fig. 3b and Supplementary Note 4). This was confirmed with a synthetic OL (see below). The three features would have the sum compositions of OL 30:0, OL 31:0 and OL 32:0, detected as [M+H]+ ions. In OL, a 3-hydroxy fatty acid is connected via an amide linkage to the ornithine amino acid that serves as the headgroup. A second fatty acid is then connected to the first via an ester linkage^35^. OL are bacteria-specific non-phosphorus glycolipids that are found in the outer membranes of selected Gram-negative bacteria^36,37^.

### *A. muciniphila* produces OL in the mouse and human gut

**Fig. 4:**
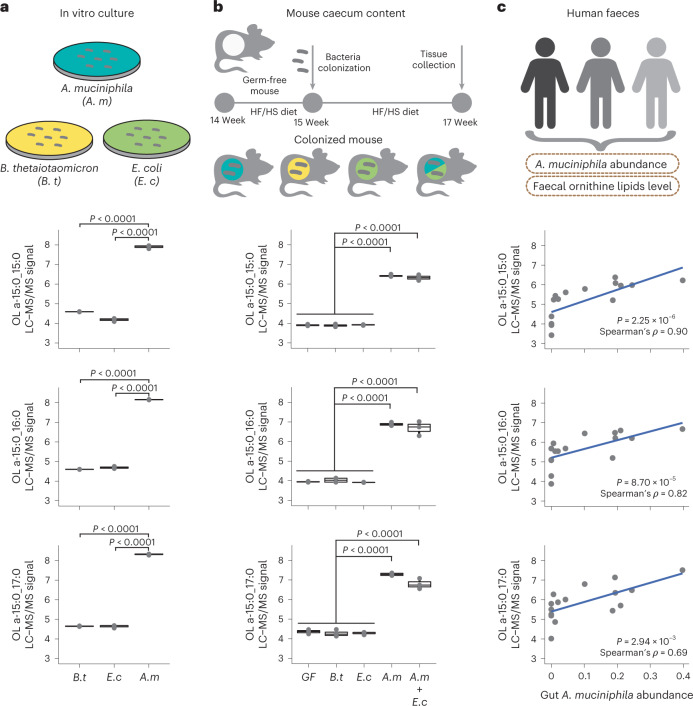
*A. muciniphila* produces OL in the mouse and human gut. **a**, OL abundance for the three major species detected in mice in cell pellets collected from *A. muciniphila* (*A. m*), *B. thetalotamicron* (*B. t*) and *E. coli* (*E. c*) grown in vitro (*n* = 3 biologically independent samples per organism). **b**, OL detected in caecal contents from gnotobiotic mice colonized with *A. muciniphila*, *B. thetaiotaomicron*, *E. coli* and *A. muciniphila* plus *E. coli* for two weeks (*n* = 3–4 mice per treatment). **c**, Detection of prominent OL species in human faecal samples is significantly correlated with *A. muciniphila* abundance as determined by two-sided Spearman correlation (*n* = 16 independent faecal samples). Box and whisker plots denote the interquartile range, median and spread of points within 1.5 times the interquartile range; data beyond the end of the whiskers are plotted individually. Statistical difference between treatment groups was tested by unpaired two-sided Welch’s *t*- test. Source data

**Extended Data Fig. 6:**
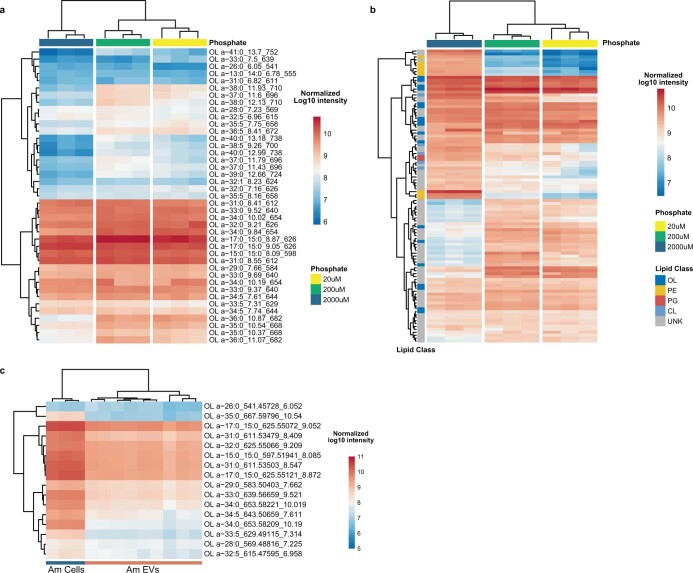
Detection of ornithine lipids (OL) in Akkermansia muciniphila. **a**, Heatmap showing relative abundance of all OL species detected in cell pellets from *A. muciniphila* grown in vitro in defined media supplemented with different levels of phosphate: 20 µM, 200 µM and 2000µM. **b**, Relative abundance of lipid features detected in cell pellets from *A. muciniphila* grown in defined media with different levels of phosphate. Top 200 most abundant lipids features are shown. **c**, Relative abundance of OL features detected extracellular vesicles (AmEVs) purified from *A. muciniphila* grown in defined medium with the comparison to *A. muciniphila* cells. Source data

*A. muciniphila* is a Gram-negative bacterium that has been associated with many beneficial effects on host metabolic health^38,39^. While previous research suggests that OL are important for microbe–host interactions^25,40^, the occurrence of these lipids in gut bacteria was not known. To test whether *A. muciniphila* produces OL, we first profiled lipids in *A. muciniphila* and two other Gram-negative species, *Bacteroides thetaiotaomicron* and *Escherichia coli* grown under anaerobic conditions. We found similarly high levels of all three targeted OL species in extracts from *A. muciniphila* but not in the other species, which were indistinguishable from the solvent blank (Fig. 4a). Since phosphate limitation triggers production of OL in some bacterial species^25^, in follow-up experiments we tested whether phosphate levels modulated abundance of OL in *A. muciniphila* grown in vitro. We examined three different levels of phosphate (0.02 mM (growth limiting), 0.2 mM (adequate) and 2 mM (excess)). LC–MS/MS analysis confirmed that OL are a dominant lipid species detected in *A. muciniphila* cell extracts regardless of the phosphate levels included in the growth media (Extended Data Fig. 6a,b). Furthermore, OL were detected in extracellular vesicles isolated from *A. muciniphila* grown in vitro (Extended Data Fig. 6c and Supplementary Note 6). These results suggest that OL are probably localized in the *A. muciniphila* outer membranes and provide insights into how these lipids may interact with the host.

We further profiled lipids produced by *A. muciniphila* colonizing the gut of gnotobiotic mice. Five groups of adult germ-free B6 mice were mono-colonized with each of the species mentioned above, bi-associated with *E. coli* and *A. muciniphila* or kept germ-free (*n* = 3–5 per group). Mice were maintained in the same HF/HS diet used for the DO study for two weeks after inoculation. LC–MS/MS analysis of caecal contents from these mice showed that only mice colonized with *A. muciniphila* had detectable levels of OL in their caecum (Fig. 4b). Altogether, these results confirm that *A. muciniphila* gut colonization is causally linked with high levels of OL.

We examined whether *A. muciniphila* colonization is associated with the presence of OL in the human gut. We analysed lipid content in a subset of faecal samples from a previously characterized cohort of old adults^41^ spanning a wide range of *A. muciniphila* relative abundances (not detectable to 39.8%). LC–MS/MS analysis of these human faecal samples detected a broader range of OL species than axenic cultures or mice colonized with *A. muciniphila*, but the levels of the three previously identified OL 15:0_15:0, OL 16:0_15:0 and OL 17:0_15:0 were all significantly correlated with *A. muciniphila* levels (Fig. 4c). Together, these results suggest that *A. muciniphila* is a major producer of OL in the mouse and human gut.

### OL modulate lipopolysaccharide (LPS)-induced cytokine responses

**Fig. 5:**
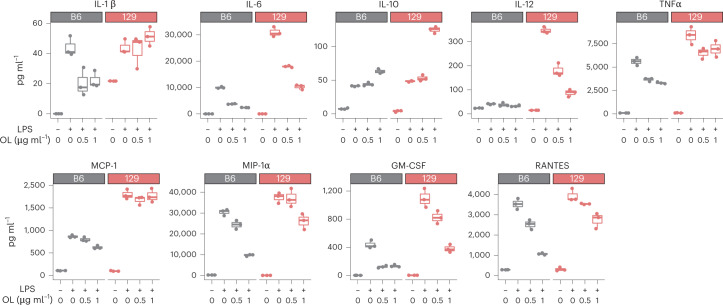
OL modulate LPS-induced production of cytokines from BMDM. Levels of IL-1β, IL-6, IL-10, IL-12, TNF-α, MCP-1, MIP-1α, GM-CSF and RANTES detected in supernatants from B6 and 129 mice BMDM stimulated with LPS (10 ng ml^−1^) and different concentrations of OL. Box and whisker plots denote the interquartile range, median and spread of points within 1.5 times the interquartile range; data beyond the end of the whiskers are plotted individually. Source data

**Extended Data Fig. 7:**
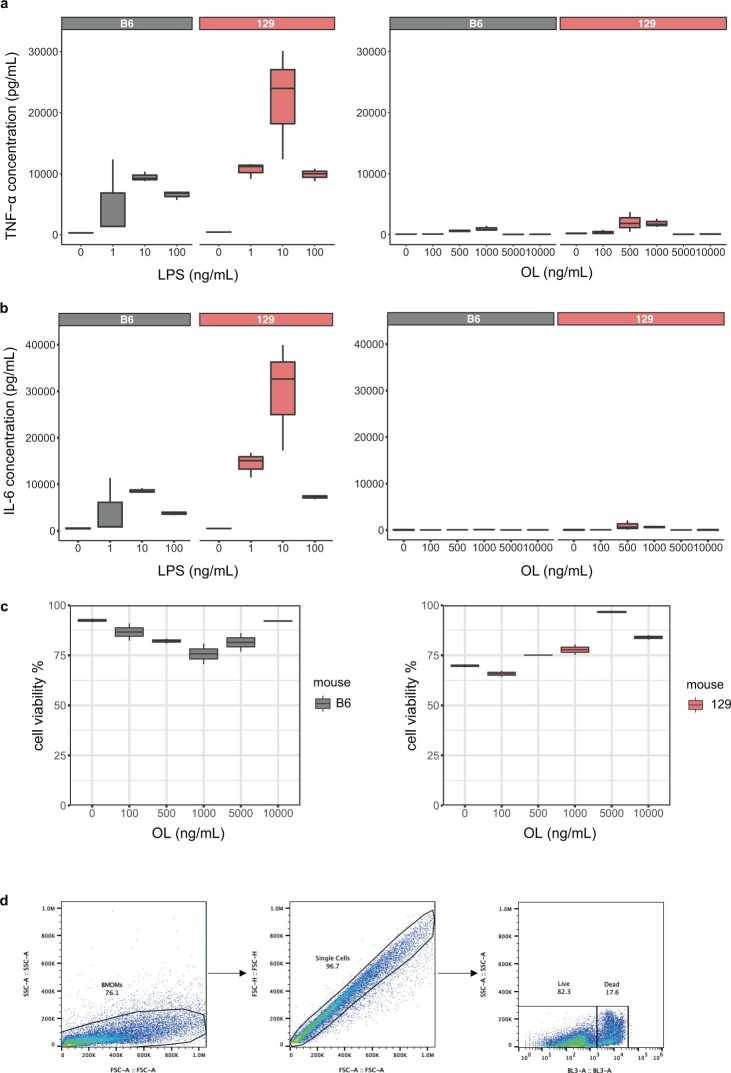
Cytokine production by BMDM. **a**,**b**, (**a**) TNF-α and (**b**) IL-6 levels detected in supernatants from BMDM cells in B6 and 129 mice treated for six hours with different concentrations of LPS or OL. **c**, Cell viability of BMDM cells in B6 and 129 mice treated for six hours with 10 ng/mL LPS and different concentrations of OL. **d**, Flow cytometry gating strategy for BMDM cell viability assays. N = 3 biological replicates/treatment group. Box and whisker plots denote the interquartile range, median and spread of points within 1.5 times the interquartile range, data beyond the end of the whiskers are plotted individually. Source data

To test whether *A. muciniphila*-derived OL elicit immune responses on the host, we first chemically synthesized the most abundant OL detected in the DO mouse gut, that is, OL_15:0_15:0. Because of the generally beneficial effects of *A. muciniphila* on host health as previously documented in both human and mouse studies, and on the basis of the structural similarity between OL and lipid A from LPS, we speculated that the OL might function as antagonists of lipid A. We examined the effects of the OL preparation in the absence and presence of LPS on cytokine production by bone-marrow-derived-macrophages (BMDM). Treatment with LPS induced a significant increase in the production of TNF-α and IL-6 by BMDM obtained from B6 and 129 mice (Extended Data Fig. 7a). In contrast, treatment with OL preparation did not stimulate significant production of TNF-α and IL-6 by these cells (Extended Data Fig. 7b), except for a modest increase at 500 ng ml^−1^ and 1,000 ng ml^−1^. However, we observed that pretreatment of macrophages with OL had an inhibitory effect on LPS-induced TNF-α and IL-6 in both B6 and 129 mice without causing significant changes in cell viability (Extended Data Fig. 7c,d). These results suggest that *A. muciniphila*-derived OL can prevent LPS-induced inflammation response. Furthermore, we measured other cytokines secreted by LPS-treated BMDM and observed that the OL preparation inhibited the production of IL-1β, MCP-1, MIP-1α, GM-CSF, IL-12 and RANTES (Fig. 5), although there were differences in the responses to LPS and OL as a function of BMDM genetic background. In addition, OL increased the levels of anti-inflammatory cytokine IL-10 in these cells (Fig. 5), suggesting that OL may modulate inflammation by altering the levels of both pro-inflammatory and anti-inflammatory cytokines. Interestingly, production of IL-12 in the presence of LPS was more than ten times higher in 129 mice than in B6 mice, and OL had a larger inhibitory effect in these mice (Fig. 5). These results indicate that *A. muciniphila-*derived OL may influence host innate immune responses and their effects may vary as a function of host genetics.

### Intestinal genes co-map with *A. muciniphila* and OL QTL

**Extended Data Fig. 8:**
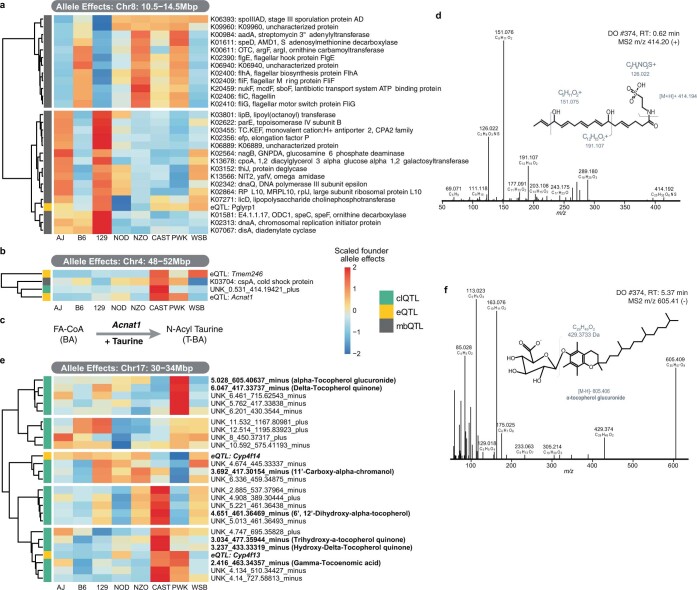
Examples of co-mapping QTL. **a**, At Chr8: 10.5-14.5 Mbp, co-mapping of gut bacterial lipopolysaccharide cholinephosphotransferase function with *Pglyrp1* eQTL was observed. **b**, At Chr4: 50 Mbp, co-mapping of an unidentified caecal feature and a local *Acnat1* eQTL was observed. **c**, The knowledge of *Acnat1* conjugating taurine to fatty acids guided the identification of the feature as an N-acyl taurine. **d**, Fragmentation pattern of identified N-acyl taurine. **e**, At Chr17: 30-34 Mbp, several unidentified features co-mapped which subsequently could be identified as tocopherols and exemplarily shown for the most significant feature alpha-tocopherol glucuronide. **f**, Fragmentation pattern of identified alpha-tocopherol glucuronide. Source data

We sought to generate regulatory maps of gene expression regulation in the small intestine and to identify overlapping SNPs associated with gut microbiome. We reasoned that identifying genes whose expression demonstrate shared genetic architecture with bacterial taxa/genes/lipids would not only narrow the list of candidate genes at each locus but would also provide invaluable insights into the biology underlying the microbe–host interactions. Furthermore, the power of expression QTL (eQTL) mapping to connect genetic polymorphism and complex traits has been well documented by others^42,43^. We profiled transcript levels in the distal small intestines of 234 DO mice using RNA-seq. We detected 8,137 transcripts with a minimum of ten counts per million (CPM) in at least 10% of DO mice. We identified 4,462 local eQTL with an average LOD score of 21.2 and 10,894 distal eQTL with an average LOD score of 7.1 (Supplementary Table 12). By comparing eQTL allele effects with those for the co-mapping mbQTL and clQTL, we identified gut microbial features and caecal lipids that were potentially co-regulated with intestinal transcripts (Extended Data Fig. 8 and Supplementary Note 7).

**Fig. 6:**
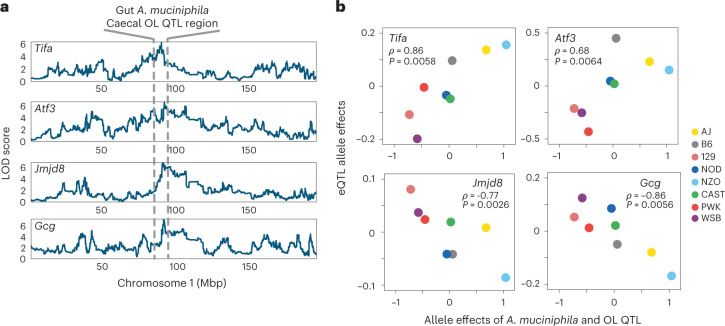
eQTL for distal small intestine (ileum) genes that co-map with *A. muciniphila* and caecal OL at Chromosome 1. **a**, QTL of *A. muciniphila*, caecal OL and eQTL for *Tifa*, *Atf3*, *Jmjd8* and *Gcg* co-map at Chr1: 90–95 Mbp. LOD score in *y* axis represents significance of QTL for each trait. **b**, Spearman correlation of allele effects between *Tifa*, *Atf3*, *Jmjd8* and *Gcg* gene eQTL and *A. muciniphila*/OL QTL. Source data

**Extended Data Fig. 9:**
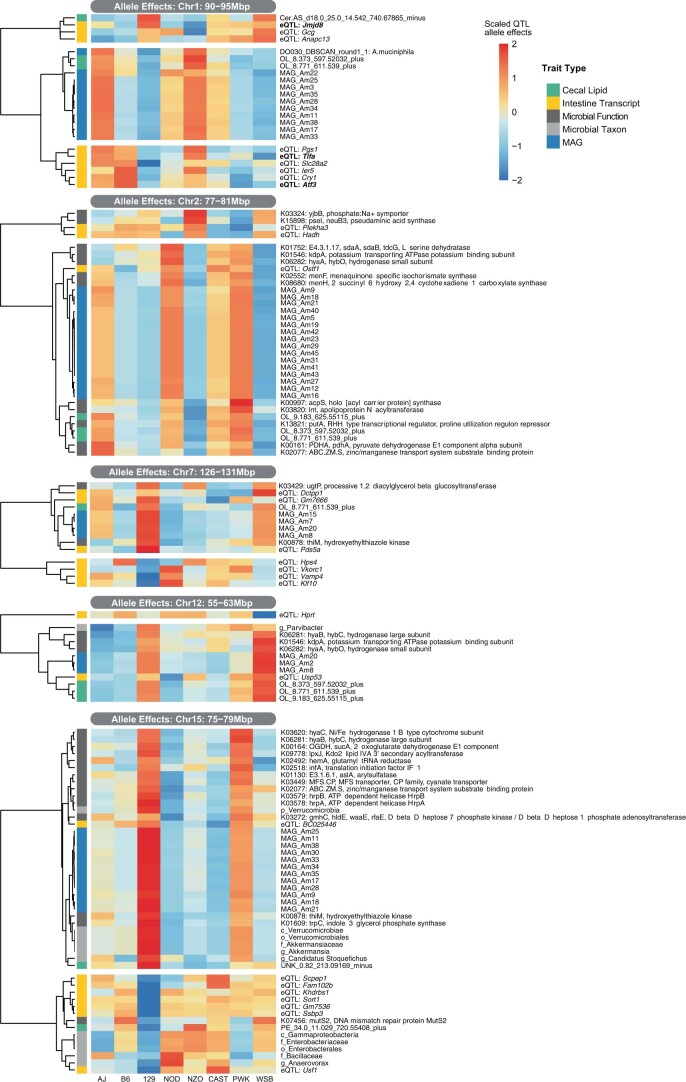
Founder allele effects on co-mapping traits associated with *A. muciniphila* levels. *A. muciniphila*, caecal OL and eQTL genes co-mapping at Chr1: 90-95 Mbp, Chr2: 77-81 Mbp, Chr7: 126-131 Mbp, Chr12: 55-63 Mbp and Chr15: 75-79 Mbp. Source data

We searched the support intervals for the five co-mapping QTL regions for *A. muciniphila* and OL (Chr1, Chr2, Chr7, Chr12 and Chr15) for candidate host genes of interest using the eQTL data. By comparing the allele effects between co-mapping eQTL and the *A. muciniphila*/OL QTL, we identified several candidate host genes whose eQTL allele effects were correlated with *A. muciniphila*/OL (Fig. 6, Extended Data Fig. 9 and Supplementary Table 13). At the Chr1 QTL region, there were four candidate genes: (1) Gene Activating transcription factor 3 (*Atf3*) had a distal eQTL at Chr1: 92.96 Mbp with QTL LOD score of 6.55. ATF3 plays an important role during host immune response events by negatively regulating the transcription of pro-inflammatory cytokines induced by the activation of toll-like receptor 4^44^. (2) The gene TRAF-interacting protein with a forkhead-associated domain (*Tifa*) had a distal eQTL at Chr1: 90.95 Mbp with LOD score of 6.19. TIFA has been reported to sense bacterial-derived heptose-1,7-bisphosphate—an intermediate in the synthesis of LPS—via a cytosolic surveillance pathway triggering the NF-kB response^45,46^. Additionally, TIFA interacts with TRAF6 to mediate host innate immune responses. (3) The gene Jumonji domain-containing protein 8 (*Jmjd8*) had a distal eQTL at Chr1: 92.14 Mbp with LOD score of 6.72. JMJD8 functions as a positive regulator of TNF-induced NF-kB signalling^47^. A recent study showed that JMJD8 is required for LPS-mediated inflammation and insulin resistance in adipocytes^48^. (4) The gene *Gcg* had a distal eQTL at Chr1: 92.36 Mbp with LOD score of 7.11. *Gcg* encodes multiple peptides including glucagon, glucagon-like peptide-1(GLP-1). GLP-1 levels are induced by a variety of inflammatory stimuli, including endotoxin, IL-1β and IL-6^49^. The finding that these genes with distal eQTL that co-map with *A. muciniphila* and OL QTL on Chr1 are involved in host immune responses to microbial-associated molecular patterns (MAMPs) such as LPS suggests that expression of these genes contributes to the regulation of host responses to OL and/or potentially modulates the abundance of *A. muciniphila*.

### Dissecting the link between *A. muciniphila* and *Atf3*

**Extended Data Fig. 10:**
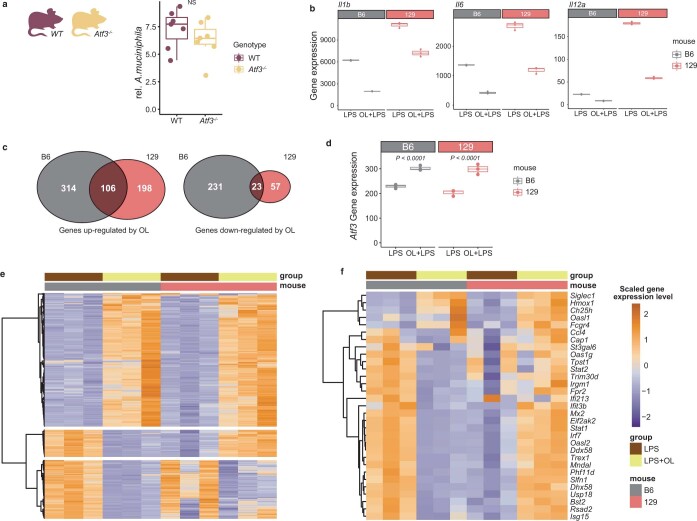
Expression of genes in BMDM treated with OL/LPS. **a**, Abundance of *A. muciniphila* in faecal pellets from *Atf3*^*-/-*^ mice and WT mice (n = 7 mice/genotype; four females, three males for both genotypes). **b**, Gene expression level of *Il1b*, *Il6* and *Il12a* from BMDM cells derived from B6 and 129 mice treated for six hours with LPS (10 ng/ml) or with LPS (10 ng/mL) and OL (1 µg/mL). N = 3 biological replicates/treatment group. **c**, Number of differentially expressed genes in BMDM derived from B6 and 129 mice. **d**, Gene expression levels of *Atf3* in BMDM from B6 and 129 mice treated for six hours with LPS (10 ng/mL) or LPS (10 ng/mL) and OL (1 µg/mL). N = 3 biological replicates/genotype/treatment group. **e**, Differentially expressed genes in BMDM from B6 and 129 mice. **f**, Previously reported ATF3 regulated genes in BMDM^50^. Impact of OL on these genes in B6 and 129 mice. Box and whisker plots denote the interquartile range, median and spread of points within 1.5 times the interquartile range; data beyond the end of the whiskers are plotted individually. Statistical difference between treatment groups was tested by two-sided Welch’s *t*- test. Source data

We investigated whether the co-mapping between *A. muciniphila*/OL QTL and *Atf3* gene eQTL could be explained by ATF3 impacting the abundance of these traits. To address this question, we measured the abundance of this taxon in wild-type (WT) mice and animals lacking the *Atf3* gene consuming HF/HS diet for four weeks. We observed that *Atf3*^−/−^ and WT mice had comparable levels of *A. muciniphila* in faeces as detected by qPCR. Abundance of *A. muciniphila* was ~15% lower in faecal samples from *Atf3*^−/−^ mice compared with wild type (*n* = 7 per genotype), yet the differences did not reach significance (Extended Data Fig. 10a). These results suggest that *Atf3* does not play a major role in *A. muciniphila* fitness. It might also act in combination with other factors, which would align with the observation that the abundance of gut *A. muciniphila* is a polygenic trait.

An alternative explanation for the observed co-mapping is that *A. muciniphila*/OL modulate expression of *Atf3*. To examine this idea, we assessed expression profiles of B6 and 129 BMDM stimulated with LPS or a combination of the OL preparation and LPS. DESeq2 analysis identified 674 genes differentially expressed in cells from B6 mice treated with OL (420 genes were upregulated and 254 genes downregulated), whereas 384 genes (304 genes were upregulated and 80 genes downregulated) were impacted by OL in BMDM derived from 129 mice. While differences in gene expression of some of the cytokines discussed above (Extended Data Fig. 10b) were consistent between genotypes, the overall overlap of differentially expressed genes between genotypes was relatively low (Extended Data Fig. 10c) and the responses to the OL varied significantly by genotype (Extended Data Fig. 10e). As mentioned above, ATF3 is a negative regulator of TLR4 signalling. We observed that OL upregulated *Atf3* expression for both B6 and 129 BMDMs (Extended Data Fig. 10d). Furthermore, a previous study^50^ identified 30 genes downregulated by ATF3 in BMDMs (B6 background). Consistent with this result, we found that OL downregulated the expression of these genes in BMDM derived from B6 mice. In contrast, we found that 18 out of these 30 genes were upregulated by OL in BMDM from 129 mice (Extended Data Fig. 10f). These results suggest that the observed co-mapping between *A. muciniphila*/OL QTL and *Atf3* eQTL could be explained by the effect of OL on *Atf3* gene expression and that increased expression of this gene may trigger distinct programmes as a function of host genotype potentially impacting immune and metabolic responses differently.

Altogether, the work supports the notion that *A. muciniphila* is the major producer of caecal OL in the distal gut and that *A. muciniphila*-produced OL can negatively regulate host LPS-induced inflammation by upregulating *Atf3* expression.

## Discussion

We applied a systems genetics approach to identify relationships between gut microbes, their encoded functions, caecal lipids and host intestinal gene expression. We identified bacterial functions influenced by host genetic variation and discovered that the bacterium *A. muciniphila* produces immunoactive OL that are detected in faecal samples from humans and mice colonized with this bacterium. *A. muciniphila* has been previously associated with host genetic variation at several loci in both mice and humans^11,12,51,52^; however, environmental conditions including diet, which is a major known determinant of microbiome composition, differ dramatically among these studies. The associations described in the present study differ from the ones previously reported in other mouse studies using different diets^12,51^. We also examined whether gut microbiome traits acted as mediator to previously published metabolic traits for the same cohort of DO mice^53^; however, no significant mediation was detected, possibly due to the limited statistical power of our study to infer the influence of the gut microbiome on complex metabolic traits.

Previous work suggested that some Gram-negative bacteria produce OL under phosphate-limiting conditions^54–56^. In contrast, we observed that OL levels were consistently high across a 100-fold phosphate level range, suggesting that phosphate is not a major driver of OL synthesis in *A. muciniphila*. Notably, a recent study showed that increased OL production by the bacterial pathogen *Pseudomonas aeruginosa* makes its cellular surface more hydrophobic, and resulted in lower virulence and higher resistance to antimicrobials and host immune defences^25^. *A. muciniphila* consumes host glycans present in the mucus layer, which is in proximity to the host epithelium. While mucin carbohydrates and amino acids serve as substrates for *A. muciniphila*, there are also soluble host defence molecules trapped in this layer that prevent invasion of microbes to the underlying mucosal epithelial cells. We speculate that membrane OL impact interactions of *A. muciniphila* with the intestinal milieu and may represent an adaptation critical to its niche and important for its interactions with the host. Development of tools to genetically manipulate *A. muciniphila* will be needed to test these hypotheses.

The inhibitory effects of OL on LPS-induced cytokines that we and others have observed^57,58^ may represent an important aspect of how *A. muciniphila* impact host physiology. Previous studies identified both natural and synthetic molecules that can inhibit TLR4-mediated LPS signalling—compounds that prevent septic shock, and have anti-inflammatory and anti-neuropathic pain activities in vivo^59^. One group of LPS antagonist molecules targeting CD14 shares structural features with *A. muciniphila* OL including a glucose unit linked to two hydrophobic chains and a basic nitrogen on C-6^60^, supporting the potential anti-inflammatory effects of OL. Although the precise mechanisms of how OL inhibit LPS signalling are unknown, our study suggests that *A. muciniphila*-derived OL may modulate inflammatory responses.

Remarkably, three host innate immunity genes—*Atf3, Tifa* and *Jmjd8*—were co-regulated with *A. muciniphila*. *Tifa* is located in the ‘cytokine-dependent colitis susceptibility locus’ (*Cdcs1*) region, a critical genetic determinant of colitis susceptibility in 129 and B6 strains^61^. TIFA is an important modifier of innate immune signalling through its regulation of TRAF proteins, leading to the activation of NF‐κB and inflammation. Considering the importance of TIFA-dependent immunity to Gram-negative bacteria^45^, and the differential effects of OL on LPS-treated BMDM from 129 and B6 strains, our results suggest that this gene could be a key player in *A. muciniphila*-OL–host interactions. Previous studies suggested that ATF3 modulates inflammatory responses by suppressing the expression of TLR4 or CCL4 in macrophages^44,62^ and revealed a critical role of microbiota in ATF3-mediated gut homoeostasis^63^. These studies showed that ATF3 negatively regulates *Il6* and *Il12* gene expression levels^44^. In line with this, we found that OL negatively influence these cytokines in LPS-treated BMDM, and their abundance is associated with the same locus that influences *Atf3* expression. Previous studies also showed that ATF3 positively regulates host expression of antimicrobial peptides^64^ and suggested that the production of OL makes the bacterium *P. aeruginosa* more hydrophobic and resistant to cationic antimicrobial peptides^25^. However, we observe neither co-mapping of *A. muciniphila* with expression of antimicrobial peptides nor pronounced differences in *A. muciniphila* colonization levels between *Atf3*^−/−^ mice and WT littermates. Instead, the co-mapping of *A. muciniphila* and *Atf3* could be explained by our findings suggesting that (1) *A. muciniphila* is a major producer of OL in the gut and (2) OL upregulate expression of this key regulator. Although the molecular mechanisms underlying these observations warrant further investigation, these results suggest that *A. muciniphila* and OL levels are linked to central players of the host immune defence system and support the predominant role of host genetics as a determinant of responses to gut microbes, in particular to *A. muciniphila*.

In summary, the work presented here links the presence of OL in the human and mouse gut with *A. muciniphila* and suggests that these lipids are key players in *A. muciniphila*–host interactions. Our work highlights the importance of bacterial functions and lipids as mediators of the influence of host genetics on the gut microbiome.

## Methods

### Animal studies

Animal care and study protocols were approved by the AAALAC-accredited Institutional Animal Care and Use Committee of the College of Agricultural Life Sciences at the University of Wisconsin-Madison (UW-Madison). All experiments with mice were performed under protocols approved by the UW-Madison Animal Care and Use Committee (Protocol number A005821 for the DO mice, Protocol number M00559 for gnotobiotic and *Atf3* KO mice).

#### DO mouse model

DO mice were obtained from the Jackson Laboratory at ~four weeks of age and maintained in the Department of Biochemistry vivarium at the UW-Madison. DO mice were allocated in waves of 100 animals, each with an equal number of males and females. All mice were maintained in a temperature (22.2 °C) and humidity (60%) controlled environment under a 12 h light/dark cycle (lights on at 6:00 and off at 18:00). All mice were fed an HF/HS diet (TD.08811, Envigo Teklad, 44.6% kcal fat, 34% carbohydrate and 17.3% protein) and received sterilized water ad libitum upon arrival at the facility. Mice were kept in the same vivarium room and were individually housed to monitor food intake and prevent cross-inoculation via coprophagy. DO mice were killed at 22–25 weeks of age. Faecal samples were collected immediately before euthanasia after a four h fast. Caecal contents and additional tissues were collected promptly after killing and all samples were immediately flash frozen in liquid nitrogen and stored at −80 °C until further processing. Other studies have been published with these mice^13,53,65,66^.

#### Gnotobiotic studies

C57BL/6J germ-free mice were bred and housed in the gnotobiotic mouse facility at the UW-Madison. Male mice were used for the ornithine lipid study. All mice were maintained in a controlled environment (22.2 °C, 60% humidity) in plastic flexible film gnotobiotic isolators under a strict 12 h light/dark cycle (lights on at 6:00 and off at 18:00) on standard chow diet (LabDiet 5021). At eight weeks of age, mice were switched to a western-style HF/HS diet (44.6% kcal fat, 34% carbohydrate and 17.3% protein) from Envigo Teklad (TD.08811) and orally gavaged with 200 µl of bacterial cultures. At two weeks after colonization, mice were euthanized and caecal contents collected.

##### DO founder mice

C57BL6J (B6) and 129S1/SvImJ (129) male mice (five weeks old) were obtained from the Jackson Laboratory. All mice were maintained in a controlled environment (22.2 °C, 60% humidity) under a strict 12 h light/dark cycle (lights on at 6:00 and off at 18:00). All mice were fed a standard chow diet (LabDiet 5021) and received sterilized water ad libitum for 1 week. At six weeks of age, all mice were euthanized to collect bone marrow cells.

#### Atf3 mouse studies

*Atf3* heterozygous mice (B6.129X1-*Atf3*^*tm1Dron*^/HaiMmnc) were obtained from the Mutant Mouse Resource and Research Center at University of North Carolina. Age- and sex-matched littermates of Atf3-deficient whole body knockout mice (*Atf3*^−*/*−^) and WT mice were generated by crossing *Atf3* heterozygous mice. Mice were maintained in a controlled environment under a strict 12 h light/dark cycle (lights on at 6:00 and off at 18:00) at 22.2 °C and 60% humidity. Animals were fed an HF/HS diet (TD.08811, Envigo Teklad, 44.6% kcal fat, 34% carbohydrate and 17.3% protein) and received sterilized water ad libitum after weaning. Faecal samples were collected at seven weeks of age.

#### Metagenomic shotgun DNA sequencing

Faecal DNA was extracted from individual pellets collected from DO mice using previously described methods^28,67^. Following DNA extraction, Illumina paired-end (PE) libraries were constructed using a previously described protocol^68^, with a modification of gel selecting DNA fragments at ~450 bp length. PE reads (2 × 125) were generated using a combination of MiSeq and HiSeq 2500 platforms.

#### Metagenomic reads processing

Raw reads were preprocessed using Fastx Toolkit (v0.0.13) as follows: (1) for demultiplexing raw samples, fastx_barcode_splitter.pl with –partial 2, mismatch 2 was used; (2) when more than one forward and reverse read file existed for a single sample (due to being run on more than one lane, more than one platform or at more than one time), read files were concatenated into one forward and one reverse read file; (3) barcodes were trimmed to form reads (fastx_trimmer -f 9 -Q 33) and (4) reads were trimmed to remove low-quality sequences (fastq_quality_trimmer -t 20 -l 30 -Q33). Following trimming, unpaired reads were eliminated from the analysis using custom Python scripts. To identify and eliminate host sequences, reads were aligned against the mouse genome (mm10/GRCm38) using bowtie2^69^ (v2.3.4) with default settings, and microbial DNA reads that did not align with the mouse genome were identified using samtools (v1.3) (samtools view -b -f 4 -f 8).

#### Metagenomic de novo assembly and gene prediction

After removing low-quality sequences and host contaminating DNA sequences, each metagenomic sample was de novo assembled into longer DNA fragments (contigs) using metaSPAdes^70^ (v3.11.1) with multiple *k*-mer sizes (metaspades.py -k 21, 33, 55, 77). Contigs shorter than 500 bp were discarded from further processing. Open reading frames (ORFs) (that is, microbial genes, also called metagenes) were predicted from assembled contigs via Prodigal^71^ (v2.6.3) using Hidden Markov Model (HMM) with default parameters. All predicted genes shorter than 100 bp were discarded from further processing. To remove redundant genes, all predicted ORFs were compared pairwise using the criterion of 95% identity at the nucleotide level over 90% of the length of the shorter ORFs via CD-HIT^72^ (v4.6.8). In each CD-HIT cluster, the longest ORF was selected as representative. This final non-redundant (NR) microbial gene set was defined as the DO gut microbiome NR gene catalogue.

#### Metagenomic annotation

Gene taxonomic annotation was performed using DIAMOND^73^ (v0.9.23) by aligning genes in the DO gut microbiome NR gene catalogue with the NCBI NR database (downloaded 21 December 2018) using default cutoffs: *e*-value <1 × 10^−3^ and bit score >50. Taxonomic assignment used the following parameters: ‘–taxonmap prot.accession2taxid.gz–taxonnodes nodes.dmp’ in DIAMOND command and was determined by the lowest common ancestor (LCA) algorithm when there were multiple alignments. Gene functional annotation was done using the KEGG orthology and links annotation (KOALA) method via the KEGG server (https://www.kegg.jp/ghostkoala/), using 2,698,820 prokaryote genus pan-genomes as reference. The bit score cut-off for *K*-number assignment was 60.

#### Microbiome trait quantification

Quantification of microbial genes was done by aligning clean PE reads from each sample with the DO gut microbiome NR gene catalogue using Bowtie2 (v2.3.4) and default parameters. RSEM^74^ (v1.3.1) was used to estimate microbial gene abundance. Relative abundances of microbial gene CPM were calculated using microbial gene expected counts divided by gene effective length, then normalized by the total sum. We focused the taxonomic analysis on bacteria since these represented the vast majority of annotated metagenes. We detected 1,927,034 total metagenes and from these, 1,636,209 were annotated as bacterial genes, 195 as archaeal genes, 17,372 as eukaryotic genes and 946 as viruses. There were also 272,312 genes that were unclassified. To obtain abundance information for microbial functions, the CPM of genes with the same KO annotation were summed together. In case there were multiple KO annotations for a single gene, we used all KO annotations. To obtain taxonomic abundance, the CPM of genes with the same NCBI taxa annotation were summed together at phylum, order, class, family and genus levels, with a minimum of ten genes per taxon.

#### MAGs reconstruction

To reconstruct bacterial genomes, we clustered assembled contigs with the density-based algorithm DBSCAN using features of two reduced dimensions of contigs 5-mer frequency and contig coverage. The binning process was performed by the pipeline Autometa^75^ (docker image: ijmiller2/autometa:docker_patch) and allowed deconvolution of taxonomically distinct microbial genomes from metagenomic sequences. The quality of reconstructed metagenomes was evaluated using CheckM^76^ (v1.1.3); genome completeness >90% and genome contamination <5% were required to assign high-quality MAGs. MAGs quantification was done by aligning all clean PE reads from each sample with MAGs from the same sample. Genome coverage was calculated using the bedtools (v2.29.2) ‘genomecov’ command, followed by normalization by library size across all samples. To further remove redundant MAGs, we clustered high-quality MAGs on the basis of whole-genome nucleotide similarity estimation (pairwise average nucleotide identity (ANI)) using Mash software^77^ (v2.2) with 90% ANI. From high-quality MAGs, we also annotated predicted ORFs from each MAG against the KEGG database and compared the functional potential encoded among different taxa. *A. muciniphila* MAG IDs are included in Supplementary Table 14.

#### Sample preparation for caecal lipidomic analysis

Caecal contents (30 ± 7.5 mg) along with 10 μl SPLASH Lipidomix internal standard mixture were aliquoted into a tube with a metal bead and 270 μl methanol (MeOH) were added for protein precipitation. Control samples comprised 30 ± 7.5 mg of bead beat-combined DO founder strain caecum (NZO, PWK, NOD, B6, 129, AJ) extracted with each batch. To each tube, 900 μl methyl tert-butyl ether (MTBE) and 225 μl of water were added as extraction solvents. All steps were performed at 4 °C on ice. The mixture was homogenized by bead beating for eight min at 25 Hz. Finally, the mixture was centrifuged for eight min at 11,000 × *g* at 4 °C, after which 240 μl of the lipophilic upper layer were transferred to glass vials and dried by vacuum centrifuge for 60 min.

The dried lipophilic extracts were re-suspended in 200 μl MeOH:toluene (9:1 v/v) per 10 mg dry weight (minimum of 100 μl) to account for varying water content in the samples. The dry weight was determined by drying down the remaining mixture including all solid parts.

#### LC–MS/MS analysis of DO mouse caecal samples

Sample analysis by LC–MS/MS was performed in randomized order on an Acquity CSH C18 column held at 50 °C (2.1 mm × 100 mm × 1.7 μm particle diameter; Waters) using an Ultimate 3000 RSLC binary pump (400 μl min^−1^ flow rate; Thermo Fisher) or a Vanquish binary pump for validation experiments. Mobile phase A consisted of 10 mM ammonium acetate in acetonitrile/H_2_O (70:30 v/v) containing 250 μl l^−1^ acetic acid. Mobile phase B consisted of 10 mM ammonium acetate in isopropanol/acetonitrile (90:10 v/v) with the same additives. Mobile phase B was initially held at 2% for two min and then increased to 30% over three min; further increased to 50% over one min and 85% over 14 min; and then raised to 95% over one min and held for seven min. The column was re-equilibrated for two min before the next injection.

DO lipid extracts (20 μl) were injected by an Ultimate 3000 RSLC autosampler (Thermo Fisher) coupled to a Q Exactive Focus mass spectrometer by a HESI II heated electrospray ionization (ESI) source. Both source and inlet capillary were kept at 300 °C. Sheath gas was set to 25 units, auxiliary gas to ten units and the spray voltage was set to 5,000 V (+) and 4,000 V (−), respectively. The MS was operated in polarity switching mode, acquiring positive and negative mode MS1 and MS2 spectra (Top2) during the same separation. MS acquisition parameters were 17,500 resolving power, 1 × 10^6^ automatic gain control (AGC) target for MS1 and 1 × 10^5^ AGC target for MS2 scans, 100 ms MS1 and 50 ms MS2 ion accumulation time, 200- to 1,600 Th MS1 and 200- to 2,000 Th MS2 scan range, 1 Th isolation width for fragmentation, stepped HCD collision energy (20, 30, 40 units), 1.0% under fill ratio and ten s dynamic exclusion.

#### QTL mapping

Genetic QTL mapping was performed using the R/qtl2 (v0.24) package^78^ which fit a linear mixed effect model that included accounting for overall genetic relationship with a random effect, that is, kinship effect. The leave one chromosome out (LOCO) method was used, which accounts for population structure without reducing QTL mapping power. For each gut microbiome trait and caecal lipidome traits, sex, days on diet and mouse cohort (wave) were used as additive covariates as described previously^13^. For gut microbiome traits and caecal lipidome traits, normalized abundance/coverage was transformed to normal quantiles. The mapping statistic reported was the log_10_ likelihood ratio (LOD score). The QTL support interval was defined using the 95% Bayesian confidence interval^78^. Significance thresholds for QTL were determined by permutation analysis (*n* = 1,000). We included 2,803 gut microbiome function traits, 197 gut microbiome taxon traits and 3,384 caecal lipid feature traits for our QTL mapping. The reported genome-wide *P* values were not adjusted for the multiple phenotypes to avoid overly declaring QTL in the initial analysis. We used genome-wide *P* < 0.05 for significant QTL and used genome-wide *P* < 0.2 to find concordant QTL mapping and hotspots.

#### Mediation analysis

Mediation analysis was carried out as previously described^79^. Mediation analysis was used to relate individual gut microbial metagenes and lipid features by scanning all 136,200 identified metagenes with at least ten CPM in 20% of the samples to all 3,963 caecal lipid features. We used the subset of animals for which both gut metagenomic and caecal lipid data were available (*n* = 221). We first defined gut microbial traits with suggestive QTL as the outcome variable; we then included candidate caecal lipid mediators as additive covariates in the suggestive mbQTL mapping model and re-ran the QTL analysis. We performed the same analysis with caecal lipid features as the outcome and gut microbial features as candidate mediators. A mediatory role was supported by a significant decrease in LOD score from the original outcome QTL. Significance of the LOD score drop for a given candidate gut microbial metagene mediator on a given caecal lipid QTL was estimated by *z*-score scaled by LOD score drop, and a conservative *z*-score ≤ −6 was recorded as a potential causal mediator. The mean of fitted distributions for a given gut bacterial taxon, for example all metagenes from *A. muciniphila* gut, was scaled to the corresponding *z*-score to evaluate the mediation significance for this gut bacterial taxon.

#### Bacterial culturing and bacterial extracellular vesicle isolation

*A. muciniphila* was grown anaerobically in defined medium (Supplementary Table 15). To test for the effects of phosphate condition, the concentration of phosphate in the medium was adjusted to 0.02, 0.2 or 2 mM. *E. coli* MS200-1 strain was grown in LC medium (10 g l^−1^ bacto-tryptone, 5 g l^−1^ bacto-yeast extract, 5 g l^−1^ NaCl). *B. thetaiotaomicron* strain VPI-5482 was grown in CMM medium. All bacterial strains were grown at 37 °C. Cells for lipid analyses from the three strains were obtained by centrifugation. Isolation of *A. muciniphila* extracellular vesicles used a previously described method^80^.

#### Human faecal samples

Stool samples were obtained from a previous study^41^. Samples were collected from participants of the Wisconsin Longitudinal Study. Briefly, participants collected stool samples directly into sterile containers, then samples were kept at ~4 °C until arrival (48 h or less) at the processing laboratory. Upon arrival, sterile straws were filled with the faecal material and stored at −80 °C as previously described^41^. 16S rRNA gene sequencing data for these samples were previously published. The use of the Wisconsin Longitudinal Study faecal samples was approved by the Institutional Review Board at UW-Madison. Consent from participants was obtained via a process involving both verbal and written components by trained interviewers, and records were archived both digitally and physically at UW-Madison. This effort did not include collection of samples from vulnerable populations or from minors.

#### Sample preparation for OL validation experiments

For caecal contents, 30 ± 6 mg caecal contents were aliquoted into a tube with a metal bead and 280 μl MeOH were added for protein precipitation. To each tube, 900 μl MTBE and 225 μl of water were added as extraction solvents. All steps were performed at 4 °C on ice. The mixture was homogenized by bead beating for eight min at 25 Hz. For bacterial cultures, ~75 μl of bacterial culture were aliquoted into a tube and 280 μl MeOH were added for protein precipitation. After the mixture was vortexed for 10 s, 900 μl MTBE were added as extraction solvent and the mixture was vortexed for ten s and mixed on an orbital shaker for six min. Phase separation was induced by adding 225 μl of water followed by 20 s of vortexing. All steps were performed at 4 °C on ice. Finally, each mixture was centrifuged for eight min at 11,000 × *g* at 4 °C, after which 240 μl of the lipophilic upper layer were transferred to glass vials and dried in a vacuum centrifuge for 60 min. The dried lipophilic extracts were re-suspended in 200 μl MeOH:toluene (9:1 v/v).

#### LC–MS/MS analysis of OL validation experiments

Sample analysis by LC–MS/MS was performed in randomized order on an Acquity CSH C18 column held at 50 °C (2.1 mm × 100 mm × 1.7 μm particle diameter; Waters) using an Ultimate 3000 RSLC binary pump (400 μl min^−1^ flow rate; Thermo Fisher) or a Vanquish binary pump. The same mobile phase and gradient as for the DO samples were used.

For the validation experiments, 10 μl of caecal or culture extract were injected by a Vanquish Split Sampler HT autosampler (Thermo Fisher) coupled to a Q Exactive HF mass spectrometer by a HESI II heated ESI source. Both source and inlet capillary were kept at 350 °C (Thermo Fisher). Sheath gas was set to 25 units, auxiliary gas to 15 units and spare gas to five units, while the spray voltage was set to 3,500 V and the S-lens RF level to 90. The MS was operated in polarity switching dd-MS2 mode (Top2), acquiring positive and negative mode MS1 and MS2 spectra during the same separation. MS acquisition parameters were 30,000 resolution, 1 × 10^6^ AGC target for MS1 and 5 × 10^5^ AGC target for MS2 scans, 100 ms MS1 and 50 ms MS2 ion accumulation time, 200 to 2,000 Th MS1 scan range, 1.0 Th isolation width for fragmentation and stepped HCD collision energy (20, 30, 40 units).

#### Lipidomic analysis

All resulting LC–MS lipidomics raw files were converted to mgf files via MSConvertGUI (ProteoWizard, Dr Parag Mallick, Stanford University) and processed using LipiDex^81^ and Compound Discoverer 2.0 or 2.1.0.398 (Thermo Fisher) for DO and validation experiments, respectively. All raw files were loaded into Compound Discoverer with blanks marked as such to generate two result files using the following workflow processing nodes: Input Files, Select Spectra, Align Retention Times, Detect Unknown Compounds, Group Unknown Compounds, Fill Gaps and Mark Background Compounds for the so called ‘Aligned’ result and solely Input Files, Select Spectra and Detect Unknown Compounds for an ‘Unaligned’ Result. Under Select Spectra, the retention time limits were set between 0.4 and 21 min, MS order as well as unrecognized MS order replacements were set to MS1. Further replacements were set to FTMS Mass Analyzer and HCD Activation Type. Under Align Retention Times, the mass tolerance was set to ten ppm and the maximum shift according to the data set to 0.6 min for the DO and 0.5 min for the validation experiments. Under Detect Unknown Compounds, the mass tolerance was also set to ten ppm, with an S/N threshold of five (DO) or three (validation), and a minimum peak intensity of 5 × 10^6^ (DO) or 1 × 10^5^ (validation).

For the DO samples, [M+H]+1 and [M−H]−1 were selected as ions and a maximum peak width of 0.75 min as well as a minimum number of scans per peak equalling seven were set. For the validation samples, [M+H]+1 and [M−H+TFA]−1 were selected as ions and a maximum peak width of 0.75 min as well as a minimum number of scans per peak equalling five were set. Lastly, for Group Unknown Compounds as well as Fill Gaps, mass tolerance was set to ten ppm and retention time tolerance to 0.2 min. For best compound selection, rules #1 and #2 were set to unspecified, while MS1 was selected for preferred MS order and [M+H]+1 as the preferred ion. For everything else, the default settings were used. Resulting peak tables were exported as Excel files in three levels of Compounds, Compound per File and Features (just Features for the ‘Unaligned’) and later saved as csv. In LipiDex’ Spectrum Searcher ‘LipiDex_HCD_Acetate’, ‘LipiDex_HCD_Plants’, ‘LipiDex_Splash_ISTD_Acetate’, LipiDex_HCD_ULCFA’ and ‘Ganglioside_20171205’ were selected as libraries for the DO, and ‘Coon_Lab_HCD_Acetate_20171229’, ‘Ganglioside_20171205’ and ‘Ornithine-Lipids_20180404’ for the validation experiments. For all searches, the defaults of 0.01 Th for MS1 and MS2 search tolerances, a maximum of one returned search result and an MS2 low mass cut-off of 61 Th were kept. Under the Peak Finder tab, Compound Discoverer was chosen as peak table type, and its ‘Aligned’ and ‘Unaligned’ results, as well as the MS/MS results from Spectrum Researcher were uploaded. Features had to be identified in a minimum of one file while keeping the defaults of a minimum of 75% of lipid spectral purity, an MS2 search dot product of at least 500 and reverse dot product of at least 700, as well as a multiplier of 2.0 for FWHM window, a maximum of 15 ppm mass difference, adduct/dimer and in-source fragment (and adduct and dimer) filtering and a maximum RT M.A.D Factor of 3.5. As post-processing in the DO, all features that were only found in one file and had no ID were deleted, and duplicates were also deleted. Peak areas of the three targeted ornithine lipid species were obtained via TraceFinder v3.3.350.0 (Thermo Fisher). Details of the lipid classes searched for in these databases with their respective adducts are shown in Supplementary Table 15. Lipids ID matching was performed at <±5 ppm between runs.

### OL synthesis

#### Chemicals and methods

All chemicals were obtained from Chem-Impex, Sigma-Aldrich, Agros Organics or TCI America. All reagents and solvents were used without further purification except for hexane, ethyl acetate and dichloromethane, which were distilled before use. Analytical thin-layer chromatography (TLC) was performed on 250 µm glass-backed silica plates with F-254 fluorescent indicator from Silicycle. Visualization was performed using UV light and iodine.

#### General instrumentation information

Nuclear magnetic resonance (NMR) spectra were recorded in deuterated solvents at 400 MHz on a Bruker-Avance spectrometer equipped with a BFO probe, and at 500 MHz on a Bruker-Avance spectrometer equipped with a DCH cryoprobe. Chemical shifts are reported in parts per million using residual solvent peaks or tetramethylsilane (TMS) as a reference. Couplings are reported in hertz (Hz). ESI–exact mass measurement (ESI–EMM) mass spectrometry data were collected on a Waters LCT instrument.

#### OL synthesis

Tridecanoic acid (compound 1, 3.2 g, 15 mmol) was dissolved in dichloromethane (150 ml, 0.1 M) in a round-bottom flask equipped with a stir bar. 1-(3-dimethylaminopropyl)-3-ethylcarbodiimide hydrochloride (EDC-HCl) (4.3 g, 22.5 mmol), 4-dimethylaminopyridine (DMAP) (273 mg, 2.25 mmol) and Meldrum’s acid (3.2 g, 22.5 mmol) were added to the flask, and the reaction was stirred overnight at room temperature. The next day, the reaction mixture was washed with 1 M HCl (3 × 75 ml), saturated NaHCO_3_ (3 × 75 ml) and brine (3 × 75 ml). The mixture was then dried over magnesium sulfate and concentrated under reduced pressure. The resultant oil was then dissolved in benzene (19 ml) in a round-bottom flask with a stir bar, and benzyl alcohol (45 mmol, 4.7 ml) was added. The reaction was heated to 95 °C for three hours and then concentrated under reduced pressure. The crude reaction mixture was purified by silica gel flash chromatography (5–10% ethyl acetate in hexane as eluent), yielding 3.6 g of compound 2 as an oil (69% yield over two steps).
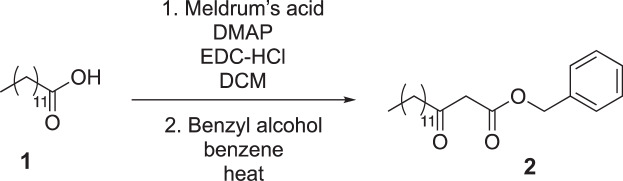


Compound 2 (3.6 g, 10.4 mmol) was added to a round-bottom flask equipped with a stir bar and dissolved in a 2:1 mixture of tetrahydrofuran (16 ml) and ethanol (8 ml). The round-bottom flask was cooled in an ice bath, and sodium cyanoborohydride (1.6 g, 26 mmol) was added to the mixture. One M aqueous HCl (26 ml, 26 mmol) was added via addition funnel, and the reaction was allowed to stir to room temperature and monitored by TLC. Upon consumption of starting material, the aqueous portion of the reaction was extracted with dichloromethane (3 × 20 ml) and combined with the organic portion. The combined organic portions were washed with brine (3 × 20 ml), dried over MgSO_4_ and concentrated under reduced pressure to yield 3.26 g of compound 3 (93% crude). The material was used without further purification.
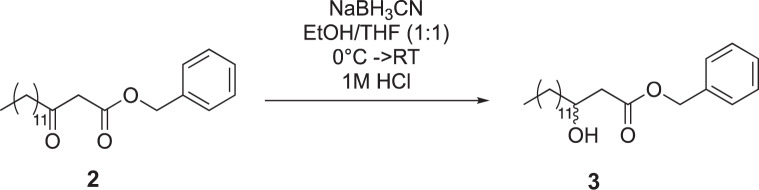


Pentadecanoic acid (1.93 g, 9 mmol) was added to a round-bottom flask equipped with a stir bar and dissolved in dichloromethane (80 ml). To the flask was added EDC-HCl (2.68 g, 14 mmol), DMAP (974 mg, 8 mmol) and compound 3 (2.78 g, 8 mmol). The reaction mixture was allowed to stir overnight at room temperature. The next day, the mixture was washed with 1 M HCl (3 × 50 ml), saturated NaHCO_3_ (3 × 50 ml) and saturated brine (3 × 50 ml). The mixture was then dried over magnesium sulfate and concentrated under reduced pressure. The crude material was purified by silica gel flash chromatography (5–10% ethyl acetate in hexane as eluent), yielding 4.3 g of compound 4 (94% isolated yield).
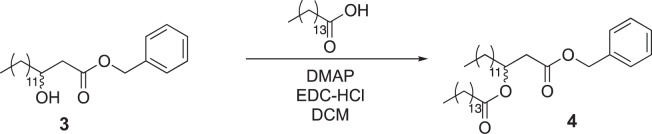


To a flame-dried round-bottom flask equipped with a stir bar was added Pd/C (798 mg, 0.75 mmol Pd). Dry dichloromethane was added to the flask to make a slurry, and the atmosphere was exchanged for nitrogen. Compound 4 (4.3 g, 7.5 mmol) was dissolved in anhydrous methanol and added to the reaction vessel. The atmosphere was then exchanged for hydrogen (balloon pressure), and the reaction was allowed to proceed overnight. The next day, the reaction was diluted with ethyl acetate and filtered over celite. The mixture was concentrated under reduced pressure to yield compound 5 as a white solid (3.5 g, 97% crude yield). The material was used without further purification.
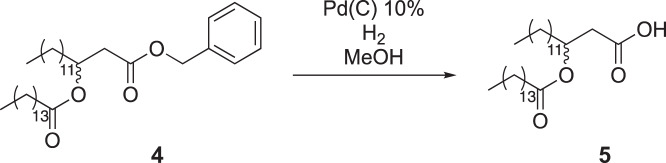


Compound 5 (256 mg 0.5 mmol) was added to a round-bottom flask equipped with a stir bar and dissolved in dimethylformamide (DMF) (5 ml). To the flask was added N,N-Diisopropylethylamine (DIPEA) (277 μl, 1.6 mmol) and hexafluorophosphate azabenzotriazole tetramethyl uronium (HATU) (216 mg, 5.5 mmol), and the mixture was stirred for 15 min. Protected ornithine (250 mg, 0.6 mmol) was added to the mixture, which was stirred at room temperature and monitored by TLC. When starting material was no longer observed by TLC, the mixture was diluted in diethyl ether (20 ml) and washed with 1 M HCl (3 × 20 ml), saturated NaHCO_3_ (3 × 20 ml) and brine (3 × 20 ml). The mixture was dried over magnesium sulfate and concentrated under reduced pressure to yield a white solid (376 mg crude). This sample was combined with an additional sample of the same crude material that appeared identical by ^1^H NMR analysis and was then purified by silica gel flash chromatography (25% ethyl acetate in hexane as eluent) to yield 131 mg of compound 6.
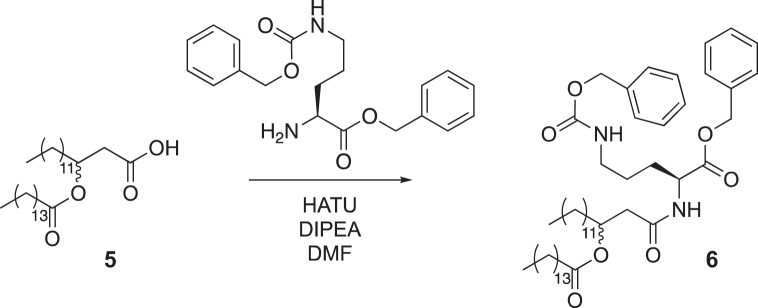


To a flame-dried round-bottom flask equipped with a stir bar was added Pd/Cn (17.0 mg, 0.16 mmol Pd). Dry dichloromethane was added to the flask to make a slurry, and the atmosphere was exchanged for nitrogen. The protected ornithine lipid (compound 6, 131 mg, 0.160 mmol) was dissolved in a mixture of 4 ml anhydrous methanol/dichloromethane (DCM) (1:1) and added to the reaction vessel. The atmosphere was then exchanged for hydrogen (balloon pressure), and the reaction was allowed to proceed overnight. The next day, the reaction was filtered over celite. The mixture was concentrated under reduced pressure to yield OL as an off-white solid (82.2 mg, 86% crude yield). Deprotected OL was identified using LC and ESI-EMM ([M]+ calculated 597.5207, measured 597.5188, 0.002 ppm) in the resultant mixture and the material was used without further purification in the experiments described herein.
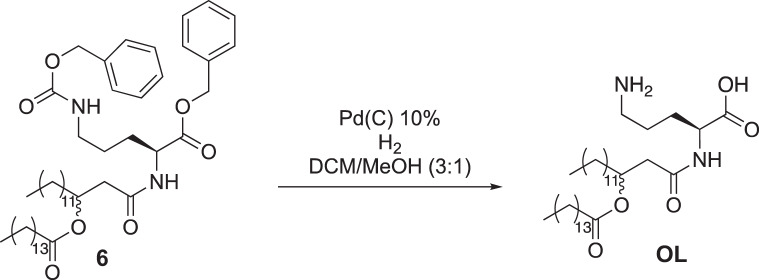


#### RNA-seq and eQTL analysis

Samples of flash-frozen distal ileum from DO mice were homogenized with Qiagen Tissuelyser (two step two min at 25 Hz, with flipping plate homogenization with five min ice incubation). Total RNA was extracted from homogenized samples using Qiagen 96 universal kit (Qiagen). RNA clean-up was performed using Qiagen RNeasy mini kit (Qiagen). DNA was removed by on-column DNase digestion (Qiagen). Purified RNA was quantified using a Nanodrop 2000 spectrophotometer and RNA fragment analyzer (Agilent). Library preparation was performed using the TruSeq Stranded mRNA sample preparation guide (Illumina). IDT unique dual indexes (UDIs), Illumina UDIs or NEXTflex UDIs were used as barcodes for each library sample. RNA sequencing was performed on an Illumina NovaSeq 6000 platform. Raw RNA-seq reads quality control was performed using Trimmomatic^82^ (v0.39) with default parameters. Genotype-free genome reconstruction and allele specific expression quantification were performed using the GBRS tool (http://churchill-lab.github.io/gbrs/). Genes with at least ten transcripts per million in at least 10% of DO mice were used for downstream analyses. For eQTL mapping, sex, RNA-seq index, RNA-seq wave and mouse cohort (wave) were used as additive covariates. eQTL analysis was otherwise the same as previously described^53^.

#### BMDM assay and cell viability measurement

Bone marrow was isolated from femur and tibia from ~six-week-old B6 and 129 mice fed with chow diet. Bone marrow cells were re-suspended into single-cell suspensions and cultured in complete DMEM medium supplemented with 10% fetal calf serum (FCS), 2 mM l-glutamine, 1% penicillin/streptomycin and 20 ng ml^−1^ mouse macrophage colony stimulating factor (M-CSF) (BioLegend) for the purpose of differentiation. BMDM cells were randomly allocated into treatment groups. BMDMs were collected at day seven and treated with LPS, OL or LPS + OL for 6 hours in media supplemented with 1% fetal bovine serum (FBS), then supernatants were collected for measurement of cytokines. For optimization, cytokine (TNF-α and IL-6) production from LPS- or OL-treated BMDM was performed using mouse TNF-α ELISA MAX Deluxe kit and mouse IL-6 ELISA MAX Deluxe kit (BioLegend), respectively. Follow-up cytokine (IL-1β, IL-6, IL-10, IL-12, MCP-1, TNF-α, MIP-1α, GM-CSF and RANTES) production assays in response to LPS + OL co-cultured BMDM were performed using Q-Plex Mouse Cytokine Screen 16-Plex (Quansys). Cell viability was determined by flow cytometry (Thermo Fisher Attune NxT) after staining with 7-amino-actinomycin D (eBioscience).

#### RNA-seq of BMDM

Frozen BMDM were homogenized with Qiagen Tissuelyser (two min at 20 Hz) and total RNA was extracted using Qiagen 96 universal kit (Qiagen). RNA clean-up was performed using Qiagen RNeasy mini kit (Qiagen). DNA was removed by on-column DNase digestion (Qiagen). Library preparation was performed using the TruSeq Stranded mRNA sample preparation guide (Illumina). RNA sequencing was performed on an Illumina NovaSeq 6000 platform. Raw RNA-seq reads quality control was performed using Trimmomatic^82^ (v0.39) with default parameters. Gene quantification was performed using RSEM^74^ (v1.3.1). DESeq2^83^ (v1.26.0) was used to identify differentially expressed genes between groups.

#### *Akkermansia*-specific qPCR for mouse faecal samples

To quantify *Akkermansia* abundance in mouse faecal samples, previously validated primers specific for *A. muciniphila* were used (forward CAGCACGTGAAGGTGGGGAC and reverse CTTGCGGTTGGCTTCAGAT)^84^. *A. muciniphila* genomic DNA isolated from a pure culture was used to generate a standard curve encompasing seven points (range: 1 ng μl^−1^–0.015625 ng μl^−1^). The PCR reaction contained SsoAdvanced Universal SYBR Green Supermix (Bio-Rad). Faecal *A. muciniphila* abundance was normalized by faecal weight.

### Data analysis and statistical analysis

All data integration and statistical analysis were performed in R (v3.6.3). Data collection and analysis were not performed blind to the conditions of the experiments. No data were excluded from the analysis. No statistical methods were used to pre-determine sample sizes, but our sample sizes are similar to those reported in previous publications^13^. Differences between groups were evaluated using unpaired two-tailed Welch’s *t*-test. Enrichment analysis was performed with Fisher’s exact test using a custom R function. Correlation analysis was performed with two-sided Spearman’s correlation using the R function ‘cor.test()’. For multiple testing, Benjamini-Hochberg false discovery rate (FDR) procedure was used to adjust *P* values. Data integration was performed using R packages dplyr (v1.0.6), tidyr (v1.1.3), reshape2 (v1.4.4) and data.table (v1.14.0). Heat maps were plotted using the R package pheatmap (v1.0.12). Other plots were created using the R packages ggplot2 (v3.3.3), gridExtra (v2.3), RcolorBrewer (v1.1-2) and ggsci (v2.9).

### Reporting summary

Further information on research design is available in the Nature Portfolio Reporting Summary linked to this article.

## Supplementary information

Supplementary Information Supplementary Notes 1–7.

Reporting Summary

Supplementary Tables 1–16.

## Data Availability

DO metagenomic WGS data are available from the Sequence Read Archive (SRA) under accession PRJNA744213. RNA-seq data are available from the Sequence Read Archive (SRA) under accession numbers PRJNA772743 and PRJNA896574. Mass spectrometry data files are available on Chorus (chorusproject.org) under accession with project ID 1681 (direct links to DO caecum lipidomics: https://chorusproject.org/anonymous/download/experiment/10cb106716da44cd924a3c73ac30083d and founder strains caecum lipidomics: https://chorusproject.org/anonymous/download/experiment/ad7566e8f45942d2ba0f579857629b55). Genotypes data and additional phenotype data associated with DO mouse are available at Dryad (10.5061/dryad.pj105). SNP associations data cc_variants.sqlite are available at https://ndownloader.figshare.com/files/18533342 and mouse genes data mouse_genes_mgi.sqlite used for QTL mapping are available at https://ndownloader.figshare.com/files/17609252. Source data are provided with this paper.

## Source data

Source Data Fig. 1 Statistical source data.
Source Data Fig. 2 Statistical source data.
Source Data Fig. 3 Statistical source data.
Source Data Fig. 4 Statistical source data.
Source Data Fig. 5 Statistical source data.
Source Data Fig. 6 Statistical source data.
Source Data Extended Data Fig. 2 Statistical source data.
Source Data Extended Data Fig. 3 Statistical source data.
Source Data Extended Data Fig. 4 Statistical source data.
Source Data Extended Data Fig. 5 Statistical source data.
Source Data Extended Data Fig. 6 Statistical source data.
Source Data Extended Data Fig. 7 Statistical source data.
Source Data Extended Data Fig. 8 Statistical source data.
Source Data Extended Data Fig. 9 Statistical source data.
Source Data Extended Data Fig. 10 Statistical source data.

## Code availability

All code used in this study is available in GitHub (https://github.com/qijunz/Zhang_DO_paper) or in the corresponding software package websites.
