# Author Correction: Genetic mapping of microbial and host traits reveals production of immunomodulatory lipids by *Akkermansia muciniphila* in the murine gut

**DOI:** 10.1038/s41564-023-01366-2

**Published:** 2023-03-27

**Authors:** Qijun Zhang, Vanessa Linke, Katherine A. Overmyer, Lindsay L. Traeger, Kazuyuki Kasahara, Ian J. Miller, Daniel E. Manson, Thomas J. Polaske, Robert L. Kerby, Julia H. Kemis, Edna A. Trujillo, Thiru R. Reddy, Jason D. Russell, Kathryn L. Schueler, Donald S. Stapleton, Mary E. Rabaglia, Marcus Seldin, Daniel M. Gatti, Gregory R. Keele, Duy T. Pham, Joseph P. Gerdt, Eugenio I. Vivas, Aldons J. Lusis, Mark P. Keller, Gary A. Churchill, Helen E. Blackwell, Karl W. Broman, Alan D. Attie, Joshua J. Coon, Federico E. Rey

**Affiliations:** 1 Department of Bacteriology, University of Wisconsin-Madison, Madison, WI USA; 2 Department of Chemistry, University of Wisconsin-Madison, Madison, WI USA; 3 IMol Polish Academy of Sciences, Warsaw, Poland; 4 ReMedy International Research Agenda Unit, IMol Polish Academy of Sciences, Warsaw, Poland; 5 Department of Biomolecular Chemistry, University of Wisconsin-Madison, Madison, WI USA; 6 Morgridge Institute for Research, Madison, WI USA; 7 Department of Biochemistry, University of Wisconsin-Madison, Madison, WI USA; 8 Departments of Microbiology, Immunology and Molecular Genetics, and Human Genetics, University of California, Los Angeles, Los Angeles, CA USA; 9 Department of Medicine, University of California, Los Angeles, Los Angeles, CA USA; 10 The Jackson Laboratory, Bar Harbor, ME USA; 11 Department of Chemistry, Indiana University, Bloomington, IN USA; 12 Department of Biostatistics and Medical Informatics, University of Wisconsin-Madison, Madison, WI USA

**Keywords:** Genetic association study, Genetic linkage study, Metagenomics, Lipidomics, Lipids

Correction to: *Nature Microbiology* 10.1038/s41564-023-01326-w. Published online 9 February 2023.

In the version of this article initially published, the author list did not display first names, as are now provided in the HTML and PDF versions of the article.

