# Supplementary material for: Genetic mapping of microbial and host traits reveals production of immunomodulatory lipids by Akkermansia muciniphila in the murine gut
Source: Nat Microbiol. Author manuscript; Available in PMC 2023 Mar 9. (PMC9981464; doi:10.1038/s41564-023-01326-w)
Supplement: Supplementary Information [file NIHMS1875196-supplement-Supplementary_Information.pdf]

Supplementary Information

Supplementary Note 1. DO metagenomic analysis

Shotgun DNA sequencing (Illumina 2X125 bp paired end reads) was performed in fecal samples collected from 297 DO mice at the end of the experiment. After removal of host DNA, each sample yielded on average 17.4 million high-quality microbial DNA paired end reads (*SD* = 7.1×10^6). All shotgun sequencing reads were used to identify potential sample mix-ups and mislabeling during the library preparation process[1]. This revealed 15 samples containing mixtures of pairs of mice that were not used for further analyses. We also removed samples with low coverage (below 10 million). We proceeded with 264 DO metagenomic samples for QTL analysis. *De novo* metagenomic assembly captured 76% of the gut microbial DNA reads into contigs (**Extended Data Fig. 2a**) and included ~1.9 million unique predicted open reading frames (i.e., metagenes) with an average length of 690 bp. In order to assess the quality of the assembled non-redundant (i.e., NR) metagenes, we compared the percentage of sequence reads that map to our NR metagene assembly *vs*. a recently published mouse gut metagenome catalog[2]. Performing *de novo* assembly resulted in a larger fraction of the reads mapping to our assembly compared to the previously published catalog, i.e., alignment rate increased from 68.97% to 83.22% (**Extended Data Fig. 2b**) consistent with the notion that the DO mouse population contains different gut microbial communities relative to the previously published mouse cohort. Annotation of NR metagenes against the Kyoto Encyclopedia of Genes and Genomes (KEGG) orthology database via GhostKOALA identified 2803 functional orthologs (i.e., KOs) across all mice. It is important to note that each KO integrate abundance information of metagenes encoding functional orthologs – potentially from different species. Among these, Carbohydrate metabolism (14.30%), Membrane transport (9.05%) and Amino acid metabolism (8.43%) represented the categories with the largest number of functions detected in the distal gut of the DO mice (**Extended Data Fig. 2c**).

We used DIAMOND, a BLAST-based taxonomic classifier tool that uses the lowest common ancestor approach to assign phylogeny to all metagenes. First, we estimated the abundance of metagenomic reads in DO mice to each metagene and removed the lower abundance metagenes by the criteria that >10 counts per million in at least 10% of samples (**Supplementary Table 1**). We then summed the counts per million values of metagenes belonging to same taxon as the abundance of that taxon (Top 20 abundant genus showed in **Extended Data Fig. 2d**). We performed metagenomic binning to obtain species level bacterial genomes. This approach allowed us to detect diverse high-abundant bacterial genomes across all samples. After removing low quality bins, we obtained 5,611 putative metagenomic-assembled genomes (MAGs) representing genomes from 6 phyla including Actinobacteria, Bacteroidetes, Firmicutes, Proteobacteria, Tenericutes and Verrucomicrobia (**Supplementary Table 2**). Among these putative MAGs, 3,218 MAGs were identified as medium-quality MAGs with genome completeness above 50% and genome contamination below 5% as estimated by CheckM tool[3] (**Extended Data Fig. 2e**). 1,871 of these were high-quality with genome completeness above 90% and genome contamination below 5%. Furthermore, by reconstructing MAGs, we not only detect abundance of gut microbes but can also identify species-level variants. For example, by estimating pairwise average nucleotide identity of 46 high-quality *A. muciniphila* MAGs, we detected two distinct clusters (**Extended Data Fig. 2f**). These contain 3849 non-synonymous SNPs, indicating potential functional differences between these two *A. muciniphila* variants. Altogether, these results underscore the power of metagenomics for describing gut microbial communities, both taxonomically and in terms of their predicted gene functions. We also estimated narrow sense heritability of gut bacterial function traits via a linear mixed model and estimated heritability distribution of bacterial functions in each pathway (**Supplementary Table 3-4**).

Supplementary Note 2. Correlation between cecal lipids and the gut microbiome

To explore potential links between cecal lipid species and the gut microbiome, we performed correlation analysis between MAGs and cecal lipids abundance. Hierarchical clustering of correlation coefficients for each MAG-lipid species pair revealed groups of lipids associated with different gut microbiome profiles (**Extended Data Fig. 5**). MAGs clustered into five major groups while cecal lipids into six groups. More than 70% of the bacterial MAGs (**Supplementary Table 8**) were positively correlated with cluster 6 of cecal lipids. Fisher's exact test analysis disclosed that monogalactosyldiacylglycerols (MGDG) and phosphatidylglycerols (PG) were enriched in lipid cluster 6 (P_{BHadj} = 1.47 x 10^{-9} and P_{BHadj} = 4.83 x 10^{-6}). These results are consistent with the notion that these lipids are highly prevalent across bacteria. In cecal lipid cluster 3, more than 90% of the lipids were phospholipids; plasmenyl-phosphatidylethanolamine (P_{BHadj} = 3.92 x 10^{-8}) and phosphatidylcholines (P_{BHadj} = 5.82 x 10^{-8}) were enriched in this cluster. The most highly positively correlated taxa with this lipid cluster were in MAG cluster 4, which contained 45 MAGs from the *Lachnospiraceae* family (67% of all MAGs in this cluster) including *Acetatifactor, Dorea, Lachnoclostridium, Roseburia*. In MAG cluster 1, 96% of the taxa were from the Bacteroidetes phylum, which were positively correlated with cecal lipid cluster 1, which were enriched in PC (P_{BHadj} = 5.39 x 10^{-7}) and ceramide [BS] (P_{BHadj} = 3.54 x 10^{-3}). Cecal lipid cluster 2 was enriched in fatty acids (P_{BHadj} = 2.37 x 10^{-25}), ceramide [NS] (P_{BHadj} = 2.30 x 10^{-12}) and ceramide [NP] (P_{BHadj} = 5.75 x 10^{-6}) and was positively correlated with both Bacteroidetes and Firmicutes MAGs. Previous studies have shown that Bacteroidetes produce sphingolipids that are important for intestinal homeostasis and symbiosis[4]. To further confirm whether the significantly associated ceramides were derived from gut bacteria, we performed LCMS/MS from cecal in germ-free (GF) and conventionally-raised (convR) mice (**Supplementary Table 9**). We matched these lipid features to the ones detected in the DO mice. We found that over 67% of matched ceramides (present in DO and GF and/or convR) were significantly higher in convR mice compared to GF mice (P_{BHadj} < 0.05, **Supplementary Table 10**), indicating these associated cecal ceramides were

derived from gut bacteria, most likely from Bacteroidetes. Altogether these results highlight potential taxa that modulate abundance of lipids in the gut.

Supplementary Note 3. Cecal lipid features are associated with host genetics

To link lipid features with the host genome, we performed QTL mapping of 3,384 mass spectral features. This resulted in 399 significant QTL for cecal lipid features (LOD > 7.6, $P_{genome\text{-}wide\text{-}adj}$ < 0.05). In order to also capture the less direct associations of bacterial-derived lipids and the host genome, we included all suggestive QTL for further analysis (LOD > 6.0). This produced a total of 3,964 cecal lipid QTL (**Fig. 2c, Supplementary Table 11**). Notably, 68% of identified lipids gave a total of 1,162 QTL while a comparable proportion of 70% of unidentified features contributed 2,802 QTL. Some of the unidentified features QTL were in the region of QTL hotspots where identified lipid features co-mapped, suggesting features may share common drivers and/or belong to the same pathway and thus potentially enable the identification of unknown cecal lipid features, as previously reported[5]. Altogether these associations provide a wealth of information offering potential molecular descriptors of the genetic regulation of the microbiome.

Supplementary Note 4. Ornithine lipids identification

The lipid features whose QTL were mediated by gut *A. muciniphila* were initially not identified by our lipidomic analysis pipeline, they appeared to be closely related to each other. The three lipid features were observed closely in retention time and their precursor *m/z* in positive mode were 597.52, 611.54, and 625.55, consistently differing by 14 Da, the weight of a $-CH_2$ group. This led us to hypothesize that they were part of a fatty acid-based lipid class. When investigating their fragmentation spectra, we noticed recurring fragments at 70.065 and 115.087 Da, matching the formulas of C_4H_8N and $C_5H_{11}N_2O$, respectively. We further identified characteristic neutral losses of water and one fatty acyl that also differed by 14 Da. Together, these findings suggest that the unidentified features could be ornithine lipids (OL). The three features would have the sum

compositions of OL 30:0, OL 31:0, and OL 32:0, detected as [M+H]+ ions. In OL, a 3-hydroxy fatty acid is connected via an amide linkage to the ornithine amino acid that serves as the headgroup. A second fatty acid is then connected to the first via an ester linkage.

Supplementary Note 5. Ornithine lipids synthesis compound characterization data

Compound **2**

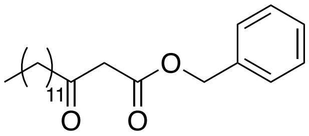

^{1}H NMR (500 MHz, Chloroform-d) δ 7.39 – 7.31 (m, 5H), 5.17 (s, 2H), 3.48 (s, 2H), 2.49 (t, J = 7.4 Hz, 2H), 1.61 – 1.52 (m, 2H), 1.25 (d, J = 5.0 Hz, 18H), 0.88 (t, J = 6.9 Hz, 3H). ^{13}C NMR (126 MHz, $CDCl_3$) δ 202.71, 167.10, 135.36, 129.04, 128.62, 128.46, 128.39, 128.23, 128.17, 125.30, 88.81, 67.10, 65.67, 53.42, 49.25, 43.10, 35.11, 31.92, 30.93, 29.65, 29.63, 29.60, 29.44, 29.35, 29.07, 29.01, 26.25, 23.46, 22.70, 14.12

Compound **3**

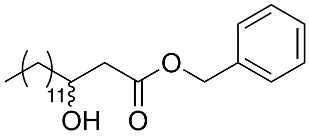

^{1}H NMR (400 MHz, Chloroform-d) δ 7.41 – 7.30 (m, 5H), 5.16 (s, 2H), 4.02 (tq, J = 7.7, 3.6 Hz, 1H), 2.89 (s, 1H), 2.62 – 2.40 (m, 2H), 1.59 – 1.37 (m, 3H), 1.37 – 1.19 (m, 18H), 0.88 (t, J = 6.7 Hz, 3H). ^{13}C NMR (126 MHz, $CDCl_3$) δ 173.02, 135.58, 128.65, 128.42, 128.28, 68.12, 67.99, 66.57, 53.43, 41.33, 36.57, 31.93, 29.68, 29.66, 29.59, 29.57, 29.52, 29.36, 25.62, 25.47, 22.70, 14.13.

Compound **4**

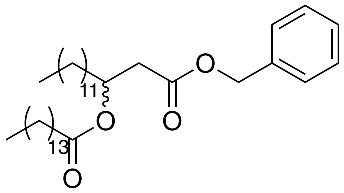

^{1}H NMR (500 MHz, Chloroform-d) δ 7.38 – 7.29 (m, 5H), 5.23 (tt, J = 7.5, 5.4 Hz, 1H), 5.11 (s, 2H), 2.67 – 2.53 (m, 2H), 2.29 – 2.11 (m, 2H), 1.66 – 1.49 (m, 5H), 1.41 – 1.19 (m, 44H), 0.88 (t, J = 6.8 Hz, 6H). ^{13}C NMR (126 MHz, $CDCl_3$) δ 173.15, 170.31, 135.79, 128.55, 128.34, 128.28, 70.28, 66.44, 39.35, 34.45, 34.06, 31.93, 29.71, 29.70, 29.68, 29.67, 29.66, 29.64, 29.55, 29.51, 29.48, 29.37, 29.36, 29.30, 29.14, 25.12, 24.99, 22.70, 22.66, 14.12. ESI-EMM: $[M+NH_4]+$ calculated 590.5139; measured 590.5139, 0.7 ppm.

Compound **5**

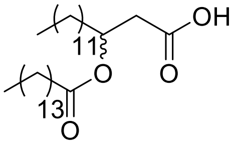

^{1}H NMR (500 MHz, Chloroform-d) δ 5.21 (p, J = 6.2 Hz, 1H), 2.60 (qd, J = 15.7, 6.3 Hz, 2H), 2.28 (t, J = 7.5 Hz, 2H), 1.61 (q, J = 7.3 Hz, 4H), 1.25 (s, 44H), 0.88 (t, J = 6.8 Hz, 6H). ^{13}C NMR (126 MHz, $CDCl_3$) δ 173.34, 70.11, 38.84, 34.50, 34.04, 31.94, 29.72, 29.71, 29.69, 29.67, 29.65, 29.56, 29.51, 29.37, 29.30, 29.15, 25.14, 25.02, 22.70, 14.12, 0.00. ESI-EMM: [M-H]- calculated 481.4262; measured 481.4297, 0.2 ppm.

Compound **6**

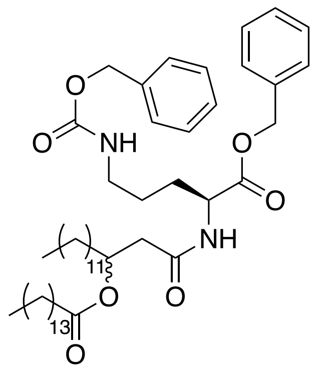

^{1}H NMR (500 MHz, Chloroform-d) δ 7.38 – 7.27 (m, 4H), 5.25 – 5.09 (m, 10H), 5.07 (s, 2H), 3.16 (q, J = 6.7 Hz, 2H), 2.50 (dq, J = 13.6, 6.9, 6.3 Hz, 2H), 2.27 (td, J = 7.4, 2.0 Hz, 2H), 1.87 (dp, J = 14.5, 4.9, 4.5 Hz, 1H), 1.74 – 1.63 (m, 1H), 1.59 (ddq, J = 17.2, 12.1, 6.0, 5.1 Hz, 4H), 1.26 (q, J = 5.5, 3.2 Hz, 44H), 0.88 (t, J = 6.9 Hz, 6H). 13C NMR (126 MHz, $CDCl_3$) δ ^{13}C NMR (126 MHz, $CDCl_3$) δ 174.35, 173.60, 173.49, 173.28, 173.27, 172.33, 172.07, 172.04, 171.19, 169.78, 169.74, 156.55, 146.18, 136.60, 135.22, 135.20, 128.65, 128.54, 128.50, 128.32, 128.08, 128.06, 71.14, 70.18, 67.28, 67.26, 66.64, 60.41, 51.89, 41.67, 41.45, 40.38, 38.94, 34.52, 34.49, 34.33, 34.14, 34.04, 31.94, 29.72, 29.71, 29.70, 29.67, 29.58, 29.53, 29.52,z 29.37, 29.33, 29.31, 29.19, 29.18, 29.15, 28.89, 28.22, 25.94, 25.88, 25.81, 25.59, 25.25, 25.22, 25.14, 25.04, 25.01, 24.99, 22.70, 14.12. ESI-EMM: [M+Na]+ calculated 843.5858; measured 843.5851, 0.8 ppm.

Supplementary Note 6. Ornithine lipids are detected in *A. muciniphila*-derived Extracellular Vesicles (AmEVs).

In Gram-negative bacteria, extracellular vesicles (EVs) are derived from the outer membrane. Previous studies suggest that bacterial EVs can penetrate the mucus barrier, get internalized in the epithelium and can also access immune cells in the lamina propria as well as play a crucial role in immunity and maintenance of gut homeostasis [6–8]. *A. muciniphila* produces extracellular vesicles that have beneficial effects on the host[9]. Animal studies showed that *A. muciniphila*-derived EVs are able to improve the intestinal barrier by increasing tight junctions[10] and ameliorating inflammation caused by colitis[11]. We examined whether OL were present in *A.*

muciniphila-derived EVs. We isolated EVs from this bacterium grown in vitro and measured the lipid features using LC-MS/MS. We found AmEVs to contain OL at levels comparable to whole *A. muciniphila* cells (**Extended Data Fig. 6b**). Ornithine lipids were among the most abundant lipid features detected in *A. muciniphila*-derived EVs with several features significantly enriched including OL_30:0, OL_31:0, and OL_32:0 which were also detected in bacterial culture, and mouse colonization studies. These results suggested that OL are likely localized in *A. muciniphila* outer membrane and provide insights into how these lipids may access the host.

Supplementary Note 7. Co-mapping QTL highlights

Co-mapping of intestinal expression QTL (eQTL) and microbiome QTL (mbQTL). One interesting example of co-mapping was observed between bacterial lipopolysaccharide cholinephosphotransferase (K07271, LicD) and host peptidoglycan recognition protein 1 (PGLYRP1) gene on Chr8. K07271 has a QTL with LOD 7.9 and a QTL peak at 12.3 Mbp on Chr8 whereas the PGLYRP1 gene has a trans-eQTL on Chr8 with LOD of 6.3 at 14.1 Mbp (**Extended Data Fig. 8a**). Notably, these co-mapping traits also share the same allele effect pattern in which 129 and NOD haplotypes have strong positive and negative associations, respectively. Correlation of QTL allele effects between these two traits was significant (Spearman's ρ = 0.96). Bacterial lipopolysaccharide cholinephosphotransferase can incorporate environmental choline into lipopolysaccharide (LPS) as phosphorylcholine (ChoP) whereas LicD affects bacterial surface structure by linking ChoP to LPS, which can in turn change susceptibility of the organism to host defense mechanisms[12]. Hosts' peptidoglycan recognition proteins (PGRPs) bind to both Gram-positive and Gram-negative bacteria and have bactericidal activity[13]. PGRPs have one or two PGRP domains with a binding site specific for bacterial peptidoglycan[14]. Furthermore, a recent study showed that *Pglyrp1*$^{-/-}$ mice have alterations in the gut microbiome and exhibit decreased responsiveness to allergic asthma[15]. While it is still not clear how changes in *Pglyrp1* gene expression impact the gut microbiome or inversely, our results suggest that

differences in Pglyrp1 gene expression are significantly associated with bacteria encoding LicD. Interestingly, we also detected several bacterial motility related genes including *fliC* (K02406), *fliG* (K02410), *flgE* (K02390), *flhA* (K02400), *fliF* (K02409) that showed opposite QTL allele effect in this region, with the 129 allele negatively associated with abundance of these traits (**Extended Data Fig. 8a**). This suggests that gut bacterial motility functions are negatively associated with host *Pglyrp1* gene expression level. Notably, two MAGs also mapped to this locus with the same allele effects as these motility related genes. These were annotated at the genus level as *Acetatifactor*. *Acetatifactor muris* was first isolated from the intestine of an obese mouse[16]. Recent studies showed that the abundance of *Acetatifactor muris* in the gut is influenced by dietary fat sources[17] and correlated with bile acids including lithocholic acid (LCA) and ursodeoxycholic acid (UDCA)[18].

Co-mapping of intestinal eQTL with cecal liplid QTL (clQTL). There were several instances in which unidentified lipid features co-mapped with other traits. Below we provide two examples: Example 1: At Chr4: ~50Mbp, we detected a strong CAST allele effect on an unidentified feature with a retention time of 0.53 minutes and an m/z of 414.19421 in positive polarity that is only shared by few other traits. Notably, among them is the local eQTL for *Acnat1*, acyl-coenzyme A amino acid N-acyltransferase 1 (**Extended Data Fig. 8b**). ACNAT1 catalyzes the addition of taurine to fatty acids to form N-acyl taurines (NAT)[19] (**Extended Data Fig. 8c**). To a lesser extent, it can also conjugate bile acids. Given this lead, the fragmentation spectrum of the feature allowed an identification as NAT 18:5;O2 (**Extended Data Fig. 8d**). Particularly intriguing was the co-mapping of microbial K03704 (Cspa, cold shock protein). CspA acts as an RNA chaperone that has been linked to the regulation of growth and stress adaptation as well as to the promotion of survival during stationary-phase. Cold shock proteins are also important for virulence in macrophages and mice. While the nature of these associations remains unknown, these results

suggest that cold shock protein may be involved in a stress response to the taurine conjugated metabolite.

Example 2: Another instance of co-mapping occurred on Chr17: ~32Mbp and included several unidentified lipid QTL (**Extended Data Fig. 8e**). We used the allele effect pattern of co-mapping traits to identify subgroups of lipid features and genes expressed in the gut having the same genetic architecture. This analysis revealed an unidentified lipid feature with an m/z of 605.41 that showed the highest significance with several genes in this region including *Cyp4f*, two genes encoding for P450 enzymes that show matching or inverse allele effects (*Cyp4f13*, *Cyp4f14*)[20]. Importantly, we were able to identify several of the unidentified features as tocopherol species, and the above-mentioned feature as alpha-tocopherol glucuronide (**Extended Data Fig. 8f**). This observation is in line with vitamin E metabolites being excreted as sulfate and glucuronide conjugates[21]. Furthermore, disruption of the *Cyp4f14* gene in mouse causes severe perturbations in vitamin E metabolism. In summary, we were able to use a combination of genomic and mass spectral data to annotate modified cecal lipid species that were unidentified after a standard database search. In several instances, co-mapping microbial QTL raised the question of an involvement of the gut microbiome in their metabolic processes.

References

1. Lobo, A. K. *et al.* Identification of sample mix-ups and mixtures in microbiome data in Diversity Outbred mice. 28.
2. Xiao, L. *et al.* A catalog of the mouse gut metagenome. *Nat. Biotechnol.* **33**, 1103–1108 (2015).
3. Parks, D. H., Imelfort, M., Skennerton, C. T., Hugenholtz, P. & Tyson, G. W. CheckM: assessing the quality of microbial genomes recovered from isolates, single cells, and metagenomes. *Genome Res.* **25**, 1043–1055 (2015).

4. Brown, E. M. *et al.* Bacteroides-Derived Sphingolipids Are Critical for Maintaining Intestinal Homeostasis and Symbiosis. *Cell Host Microbe* **25**, 668-680.e7 (2019).

5. Linke, V. *et al.* A large-scale genome–lipid association map guides lipid identification. *Nat. Metab.* **2**, 1149–1162 (2020).

6. Cañas, M.-A. *et al.* Outer Membrane Vesicles from the Probiotic Escherichia coli Nissle 1917 and the Commensal ECOR12 Enter Intestinal Epithelial Cells via Clathrin-Dependent Endocytosis and Elicit Differential Effects on DNA Damage. *PLOS ONE* **11**, e0160374 (2016).

7. Ahmadi Badi, S. *et al.* Microbiota-Derived Extracellular Vesicles as New Systemic Regulators. *Front. Microbiol.* **8**, 1610 (2017).

8. Behrouzi, A. *et al.* Comparative study of pathogenic and non-pathogenic Escherichia coli outer membrane vesicles and prediction of host-interactions with TLR signaling pathways. *BMC Res. Notes* **11**, 539 (2018).

9. Ashrafian, F. *et al.* Akkermansia muciniphila-Derived Extracellular Vesicles as a Mucosal Delivery Vector for Amelioration of Obesity in Mice. *Front. Microbiol.* **10**, 2155 (2019).

10. Chelakkot, C. *et al.* Akkermansia muciniphila-derived extracellular vesicles influence gut permeability through the regulation of tight junctions. *Exp. Mol. Med.* **50**, e450–e450 (2018).

11. Kang, C. *et al.* Extracellular Vesicles Derived from Gut Microbiota, Especially Akkermansia muciniphila, Protect the Progression of Dextran Sulfate Sodium-Induced Colitis. *PLoS ONE* **8**, e76520 (2013).

12. Lysenko, E. *et al.* The position of phosphorylcholine on the lipopolysaccharide of Haemophilus influenzae affects binding and sensitivity to C-reactive protein-mediated killing. *Mol. Microbiol.* **35**, 234–245 (2000).

13. Tydell, C. C., Yount, N., Tran, D., Yuan, J. & Selsted, M. E. Isolation, Characterization, and Antimicrobial Properties of Bovine Oligosaccharide-binding Protein. *J. Biol. Chem.* **277**, 19658–19664 (2002).

14. Royet, J. & Dziarski, R. Peptidoglycan recognition proteins: pleiotropic sensors and effectors of antimicrobial defences. *Nat. Rev. Microbiol.* **5**, 264–277 (2007).

15. Banskar, S. *et al.* The Pglyrp1 -Regulated Microbiome Enhances Experimental Allergic Asthma. *J. Immunol.* **203**, 3113–3125 (2019).

16. Pfeiffer, N. *et al.* Acetatifactor muris gen. nov., sp. nov., a novel bacterium isolated from the intestine of an obese mouse. *Arch. Microbiol.* **194**, 901–907 (2012).

17. Just, S. *et al.* The gut microbiota drives the impact of bile acids and fat source in diet on mouse metabolism. *Microbiome* **6**, 134 (2018).

18. Kübeck, R. *et al.* Dietary fat and gut microbiota interactions determine diet-induced obesity in mice. *Mol. Metab.* **5**, 1162–1174 (2016).

19. Reilly, S. *et al.* A peroxisomal acyltransferase in mouse identifies a novel pathway for taurine conjugation of fatty acids. *FASEB J.* **21**, 99–107 (2007).

20. Bardowell, S. A., Duan, F., Manor, D., Swanson, J. E. & Parker, R. S. Disruption of Mouse Cytochrome P450 4f14 (Cyp4f14 Gene) Causes Severe Perturbations in Vitamin E Metabolism. *J. Biol. Chem.* **287**, 26077–26086 (2012).

21. Schmölz, L. Complexity of vitamin E metabolism. *World J. Biol. Chem.* **7**, 14 (2016).
