# Supplementary material for: Genetic mapping of microbial and host traits reveals production of immunomodulatory lipids by Akkermansia muciniphila in the murine gut
Source: Nat Microbiol. 2023 Feb 9;8(3):424–40. doi: 10.1038/s41564-023-01326-w (PMC9981464; doi:10.1038/s41564-023-01326-w)
Supplement: Supplementary file 2 [file 41564_2023_1326_MOESM2_ESM.pdf]

Corresponding author(s): Federico E. Rey
Last updated by author(s): Dec 30, 2022

Reporting Summary

Nature Portfolio wishes to improve the reproducibility of the work that we publish. This form provides structure for consistency and transparency in reporting. For further information on Nature Portfolio policies, see our Editorial Policies and the Editorial Policy Checklist.

Statistics

For all statistical analyses, confirm that the following items are present in the figure legend, table legend, main text, or Methods section.

n/a	Confirmed
☐	☒ The exact sample size (*n*) for each experimental group/condition, given as a discrete number and unit of measurement
☐	☒ A statement on whether measurements were taken from distinct samples or whether the same sample was measured repeatedly
☐	☒ The statistical test(s) used AND whether they are one- or two-sided<br>*Only common tests should be described solely by name; describe more complex techniques in the Methods section.*
☐	☒ A description of all covariates tested
☐	☒ A description of any assumptions or corrections, such as tests of normality and adjustment for multiple comparisons
☐	☒ A full description of the statistical parameters including central tendency (e.g. means) or other basic estimates (e.g. regression coefficient) AND variation (e.g. standard deviation) or associated estimates of uncertainty (e.g. confidence intervals)
☐	☒ For null hypothesis testing, the test statistic (e.g. *F*, *t*, *r*) with confidence intervals, effect sizes, degrees of freedom and *P* value noted<br>*Give P values as exact values whenever suitable.*
☐	☒ For Bayesian analysis, information on the choice of priors and Markov chain Monte Carlo settings
☐	☒ For hierarchical and complex designs, identification of the appropriate level for tests and full reporting of outcomes
☐	☒ Estimates of effect sizes (e.g. Cohen's *d*, Pearson's *r*), indicating how they were calculated

Our web collection on statistics for biologists contains articles on many of the points above.

Software and code

Policy information about availability of computer code

Data collection	Cecal lipids. Twenty microliters of lipid extract were injected by an Ultimate 3000 RSLC autosampler (Thermo Scientific) coupled to a Q Exactive Focus mass spectrometer run by Tune software.
Data analysis	Metagenomic raw reads were preprocessed using Fastx Toolkit (ver. 0.0.13). To identify and eliminate host sequences, reads were aligned against the mouse genome (mm10/GRCm38) using bowtie2 (ver. 2.3.4) with default settings and microbial DNA reads that did not align to the mouse genome were identified using samtools (ver. 1.3). Metagenomic sample was de novo assembled into longer DNA fragments (contigs) using metaSPAdes (ver. 3.11.1) with multiple k-mer sizes (metaspades.py -k 21, 33, 55, 77). Open reading frames (ORFs) were predicted from assembled contigs via Prodigal (ver. 2.6.3). Predicted ORFs were compared pairwise using criterion of 95% identity at the nucleotide level over 90% of the length of the shorter ORFs via CD-HIT (ver. 4.6.8). Gene taxonomic annotation was performed using DIAMOND (ver. 0.9.23) by aligning genes in the DO gut microbiome NR gene catalog to NCBI NR database (downloaded 2018-12-21). Taxonomic assignment used the following parameters: "--taxonmap prot.accession2taxid.gz --taxonnodes nodes.dmp" in DIAMOND command and was determined by the LCA (Lowest Common Ancestor) algorithm when there were multiple alignments. Gene functional annotation was done using KEGG Orthology and Links Annotation (KOALA) method via the KEGG server (https: //www.kegg.jp/ghostkoala/), using 2,698,820 prokaryotes genus pan-genomes as reference. Quantification of microbial genes was done by aligning clean PE reads from each sample to the DO gut microbiome NR gene catalog using Bowtie2 (ver. 2.3.4) and RSEM (ver. 1.3.1) was used to estimate microbial gene abundance. The binning of MAGs process was performed by the pipeline Autometa (docker image: ijmiller2/autometa:docker_patch). The quality of reconstructed metagenomes was evaluated using CheckM (ver. 1.1.3). Clustering high-quality MAGs based on whole-genome nucleotide similarity estimation (pairwise ANIs) was performed by Mash software (ver. 2.2) with 90% ANI.<br>The resulting LC-MS lipidomics raw files were converted to mgf files via MSConvertGUI (ver 3.0.18205, ProteoWizard, Dr. Parag Mallick, Stanford University) and processed using Compound Discoverer 2.0 (Thermo Fisher Scientific) and an in-house developed open source software suite, LipiDex. The quantification of the internal standard was obtained through TraceFinder 4.0 (Thermo Fisher Scientific).

Data integration and statistical analysis were performed in R (ver. 3.6.3). Data integration was performed using R packages dplyr (ver. 1.0.6), tidyr (ver. 1.1.3), reshape2 (ver. 1.4.4), data.table (ver. 1.14.0). Heatmap plots were performed using R package pheatmap (ver. 1.0.12). Other plots were performed using R packages ggplot2 (ver. 3.3.3), gridExtra (ver. 2.3), RcolorBrewer (ver. 1.1-2), ggsci (ver. 2.9).
Genetic QTL mapping was performed using the R/qtl2 (ver. 0.24) package74 which fit a linear mixed effect model that included accounted for overall genetic relationship with a random effect, i.e., kinship effect. The Leave One Chromosome Out (LOCO) method was used, which accounts for population structure without reducing QTL mapping power. For each gut microbiome trait, sex, days on diet, and mouse cohort (wave) were used as additive covariates. For cecum lipidome traits, sex and mouse cohort (wave) were used as additive covariates. For gut microbiome traits and cecum lipidome traits, normalized abundance/coverage was transformed to normal quantiles. Significance thresholds for QTL were determined by permutation analysis using scan1perm() function from R/qtl2. Allele effects for each QTL were generated using the scan1blup() function from R/qtl2. SNP associations were performed using the scan1snps() function from R/qtl2.
All codes for metagenomic analysis can be found at https://github.com/qijunz/DO_metagenomics. All code for plotting and visualization can be found at https://github.com/qijunz/Zhang_DO_paper.

For manuscripts utilizing custom algorithms or software that are central to the research but not yet described in published literature, software must be made available to editors and reviewers. We strongly encourage code deposition in a community repository (e.g. GitHub). See the Nature Portfolio guidelines for submitting code & software for further information.

Data

Policy information about availability of data

All manuscripts must include a data availability statement. This statement should provide the following information, where applicable:

- Accession codes, unique identifiers, or web links for publicly available datasets
- A description of any restrictions on data availability
- For clinical datasets or third party data, please ensure that the statement adheres to our policy

DO metagenomic WGS data are available from the Sequence Read Archive (SRA) under accession PRJNA744213. RNA-Seq data are available from the Sequence Read Archive (SRA) under accession PRJNA772743. Mass spectrometry data files are available on Chorus (chorusproject.org) under accession with project ID 1681. Genotypes and additional phenotype data associated with the DO mouse population have been deposited with Dryad (doi:10.5061/dryad/pj105; data files: Attie Islet eQTL data). Genotyping used for Mouse Universal Genotyping Array (GigaMUGA; 143,259 markers). SNP associations were performed accessing variants from the database cc_variants.sqlite (available from https://ndownloader.figshare.com/files/18533342) and gene from the database mouse_genes_mgi.sqlite (available from https://ndownloader.figshare.com/files/17609252). KEGG database was downloaded from https://www.genome.jp/kegg/ at 2018-12-21.
All code used in this study is available in GitHub (https://github.com/qijunz/Zhang_DO_paper) or on the corresponding software packages websites.

Human research participants

Policy information about studies involving human research participants and Sex and Gender in Research.

Reporting on sex and gender	Samples used for our study included 11 male and 5 female.
Population characteristics	We used fecal specimens from a cohort of previously analyzed samples obtained from adults in their mid-seventies (n=408), participating in the Wisconsin Longitudinal Study (WLS). WLS is based on a one-third sample of all 1957 Wisconsin high school graduates (N=10,317) as well as selected siblings and spouses. Samples used in the present study were selected based on their Akkermansia muciniphila carrier status (reported in ref 43), low, intermediate and high.
Recruitment	The current study did not involve recruitment of participants, we used previously collected/analyzed samples. Graduates originally enrolled with an in-person questionnaire upon graduating high school in 1957, which was followed by data collection in 1964, 1975, 1992, 2004, and 2011. Siblings were surveyed in 1977, 1994, 2005, and 2011; spouses were surveyed in 2004 or 2006.
Ethics oversight	WLS data collection was approved by the Institutional Review Board (IRB) at the University of Wisconsin-Madison (2014-1066, 2015-0955).

Note that full information on the approval of the study protocol must also be provided in the manuscript.

Field-specific reporting

Please select the one below that is the best fit for your research. If you are not sure, read the appropriate sections before making your selection.

☒ Life sciences ☐ Behavioural & social sciences ☐ Ecological, evolutionary & environmental sciences

For a reference copy of the document with all sections, see nature.com/documents/nr-reporting-summary-flat.pdf

Life sciences study design

All studies must disclose on these points even when the disclosure is negative.

Sample size	A sample size of five waves of 100 DO mice each (n=500 mice total) was generated and previously described. Lipidomics assays were successfully completed for 381 samples, metagenomic analysis for 264 samples and intestinal RNAseq for 234 samples.

For bacterial culture experiment, a sample size of n=3 was chosen for each strain based on previous experience with the minimum samples size required for MS validation experiments.
For germ-free mice colonization experiment, a sample size of n=4 was chosen for each treatment group including negative control group based on previous experience with the minimum samples size required for MS validation experiments.
For bone marrow-derived macrophages experiment, a sample size of n = 9 was chosen for each treatment group (n=3 biological replicates x n=3 technical replicates).
For A. muciniphila extracellular vesicles, a sample size of n =9 was chosen (n=3 biological replicates x n=3 technical replicates).
Sample size calculation was not performed, but deemed sufficient for the purpose upon observing statistical significance in data analysis.
For Atf3 mouse experiment, a sample size of n =7 was chosen for Atf3-deficient whole body knockout mice (Atf3-/-) and littermate wild type (WT) mice.

Sample size was chosen based on pilot experiments to meet statistical significance.

Data exclusions

No data were excluded from the analysis.

Replication

The DO is an outcross population, thus, there is no biological replication of genetically identical animals. However, there is replication of genotypes at specific loci, which enables to link phenotype with genotype, as in a human GWAS studies.

Assays with bone marrow-derived macrophages were repeated at least twice. Gnotobiotic studies included built in replication (e.g., Akkermansia muciniphila monoassociation, or E. coli + Akkermansia muciniphila). All other findings were replicated at least twice.

Randomization

DO mice were allocated by waves of 100 each with an equal number of males and females. Other mice were randomly allocated into experimental groups.

Samples were randomized into batches for sample preparation and randomized again for running on the LC-MS system.

BMDM cells were randomly allocated into treatment groups.

Blinding

Blinding was not relevant to this study. All group assignment and condition were apparent to investigators.

Reporting for specific materials, systems and methods

We require information from authors about some types of materials, experimental systems and methods used in many studies. Here, indicate whether each material, system or method listed is relevant to your study. If you are not sure if a list item applies to your research, read the appropriate section before selecting a response.

Materials & experimental systems

n/a	Involved in the study
☒	☐ Antibodies
☒	☐ Eukaryotic cell lines
☒	☐ Palaeontology and archaeology
☐	☒ Animals and other organisms
☒	☐ Clinical data
☒	☐ Dual use research of concern

Methods

n/a	Involved in the study
☒	☐ ChIP-seq
☐	☒ Flow cytometry
☒	☐ MRI-based neuroimaging

Animals and other research organisms

Policy information about studies involving animals; ARRIVE guidelines recommended for reporting animal research, and Sex and Gender in Research

Laboratory animals

Equal numbers of male and female Diversity Outbred (DO) mice and eight founder strains (C57BL6J (B6), A/J (A/J), 129S1/SvImJ (129), NOD/ShiLtJ (NOD), NZO/HLtJ (NZO), CAST/EiJ (CAST), PWK/PhJ (PWK), and WSB/EiJ (WSB)) were all obtained from the Jackson Labs. All DO mice were housed within the vivarium at the Biochemistry Department, University of Wisconsin-Madison, and maintained on a Western-style high-fat/high-sucrose (HF/HS) diet (44.6% kcal fat, 34% carbohydrate and 17.3% protein) from Envigo Teklad (TD.08811) for 16 weeks. All mice were maintained in a temperature (22.2°C) and humidity (60%) controlled environment on a 12 h light/dark cycle (lights on at 6 AM and off at 6 PM), and provided water ad libitum. At ~22 weeks of age, mice were euthanized following a 4 hr fast.

Germ-free C57BL6J (B6) male mice were used for colonization experiment. Mice were housed at 22.2°C and 60% humidity. All mice were maintained in a controlled environment in plastic flexible film gnotobiotic isolators under a strict 12h light cycle and received sterilized water and standard chow (LabDiet 5021) ad libitum for 8 weeks. Then all mice were switched to a Western-style high-fat/high-sucrose (HF/HS) diet (44.6% kcal fat, 34% carbohydrate and 17.3% protein) from Envigo Teklad (TD.08811) for 2 weeks before bacteria colonization. After 2 weeks bacteria colonization, all mice were euthanized.

C57BL6J (B6) male mice and 129S1/SvImJ (129) male mice at 5 weeks of age were obtained from the Jackson Labs. Mice were housed at 22.2°C and 60% humidity. All mice were maintained in a controlled environment under a strict 12h light cycle and received sterilized water and standard chow (LabDiet 5021) for one week. At 6 weeks of age, all mice were euthanized to collect bone marrow cells.

	Atf3 heterozygous mice (B6.129X1-Atf3tm1Dron/HaiMmnc) were obtained from Mutant Mouse Resource and Research Center (MMRRC) at UNC. Age- and sex- matched littermates of Atf3-deficient whole body knockout mice (Atf3-/-) and wild type (WT) mice were generated by crossing Atf3 heterozygous mice. Mice were housed at 22.2°C and 60% humidity. All mice were fed a high-fat high-sucrose diet (TD.08811, Envigo Teklad, 44.6% kcal fat, 34% carbohydrate, and 17.3% protein) ad libitum after weaning. Fecal samples were collected at 7 weeks of age.
Wild animals	The study didn't involve wild animals.
Reporting on sex	We used equal number of males and females for DO mice and Atf3-/- mice.<br><br>We used only male mice for gnotobiotic mono- colonization and BMDM experiments. We didn't performed sex-based analysis for gnotobiotic mono- colonization and BMDM experiments.
Field-collected samples	The study didn't involve samples collected from the field.
Ethics oversight	All experiments involving mice were preapproved by an AAALAC-accredited institutional Animal Care and Use Committee of the College of Agriculture and Life Sciences (CALS) at the University of Wisconsin-Madison. The CALS Animal Care and Use Protocol number associated with the study is A005821, A. D. Attie, Principal Investigator. All experiments involving gnotobiotic mice were performed under protocols approved by the University of Wisconsin-Madison Animal Care and Use Committee (Protocol number M00559).

Note that full information on the approval of the study protocol must also be provided in the manuscript.

Flow Cytometry

Plots

Confirm that:

☒ The axis labels state the marker and fluorochrome used (e.g. CD4-FITC).

☒ The axis scales are clearly visible. Include numbers along axes only for bottom left plot of group (a 'group' is an analysis of identical markers).

☒ All plots are contour plots with outliers or pseudocolor plots.

☒ A numerical value for number of cells or percentage (with statistics) is provided.

Methodology

Sample preparation	Bone marrow cells were resuspended into single-cell suspension and cultured in complete DMEM medium supplemented with 10% FCS, 2mM l-glutamine, 1% penicillin/streptomycin, and 20 ng/ml mouse M-CSF. BMDMs were harvested at day 7 and were treated with LPS, or OL or LPS+OL for 6 hours in media supplemented with 1% FBS.
Instrument	ThermoFisher Attune NxT
Software	Prism 9
Cell population abundance	We have not sorted cells in the study.
Gating strategy	All samples were initially gated using forward scatter and side scatter to identify events corresponding to cells, and then using forward scatter height vs. area to enrich for single cells, next alive cells were selected by negativity for viability dye.

☒ Tick this box to confirm that a figure exemplifying the gating strategy is provided in the Supplementary Information.
